# The superior colliculus gates dopamine responses to conditioned stimuli in visual classical conditioning

**DOI:** 10.1038/s41467-026-72167-4

**Published:** 2026-04-29

**Authors:** Yan-Feng Zhang, Jean-Philippe Dufour, Peter Zatka-Haas, Peter Redgrave, Melony J. Black, Armin Lak, Ed Mann, Stephanie J. Cragg, Wickliffe C. Abraham, John NJ Reynolds

**Affiliations:** 1 Department of Anatomy and the Brain Health Research Centre, Brain Research New Zealand, University of Otago, Dunedin, New Zealand; 2 Department of Clinical and Biomedical Sciences, University of Exeter Medical School, Hatherly Laboratories, Exeter, UK; 3 Department of Physiology, Anatomy and Genetics, University of Oxford, Oxford, UK; 4 Department of Psychology, University of Sheffield, Sheffield, UK; 5 Department of Psychology and the Brain Health Research Centre, University of Otago, Dunedin, New Zealand

**Keywords:** Classical conditioning, Cellular neuroscience

## Abstract

In classical Pavlovian conditioning, it is well-established that midbrain dopamine neurons respond to conditioned stimuli (CS) that predict a reward. However, how the dopamine neurons associate a neutral CS to a reward remains unknown. Here, we show that in male rats and in both male and female mice, the superior colliculus (SC) develops neuronal responses to a visual CS during conditioning, which in turn drive the responses of dopamine neurons. Visual responses in the SC were only potentiated when a behaviorally meaningful time interval separated the visual stimulus and reward. Potentiation also required the convergence of visual, dopamine and serotonin inputs to the SC. Importantly, blocking potentiation of the visual response was sufficient to suppress the dopamine responses following a CS. These results reveal a mechanism for how the brain forms associations between unconditioned stimuli and behaviorally meaningful visual information during classical conditioning.

## Abstract (web summary)

Neural mechanisms underlying the ability to identify salient events are not fully understood. This study identifies the superior colliculus as a key brain region that detects the salience of visual cues and triggers dopamine release during learning, uncovering a core circuit mechanism underlying classical conditioning.

## Introduction

The ability to identify salient events as predictive of subsequent reward or danger is critical for survival. Classical conditioning, described by Pavlov more than a century ago^1^, is believed to be underpinned by brain mechanisms that associate a neutral sensory event with a reward. Much evidence implicates the phasic activity of midbrain dopamine neurons following a sensory event as reflecting processes of classical conditioning^2,3^. Thus, after repeated pairing with an unconditioned appetitive stimulus (US), a neutral stimulus becomes conditioned, and the dopamine neurons in midbrain fire burst activity following the conditioned stimulus (CS) to reflect that a reward is predicted^2,4^. For example, in visual classical conditioning, dopamine neurons start to fire in bursts at about 100 ms after a visual cue is applied to the eyes, and this dopamine response is critical for animals to learn the salience of the visual cues. But where does the formation of the US–CS association take place? The midbrain dopamine neurons do not receive primary visual input. In addition, despite the marked heterogeneity in dopamine neuron firing patterns^5,6^, dopamine neurons show a unified response to visual stimuli at individual cell bodies^7,8^ in visual classical conditioning. Thus, it is possible that dopamine neurons receive input from another brain area that itself develops the conditioned response to a visual CS.

To be the upstream brain structure that drives the dopamine response to a CS, a candidate brain structure should display a conditioned response that correlates with the presence or absence of reward. Specifically, this response should increase when the CS is associated with the US and reduce when the US is removed. Additionally, these conditioned responses should occur consistently at a shorter latency than the dopamine neuron responses, with a latency difference suitable for the candidate brain structure to convey its output to the dopamine neurons. Furthermore, the conditions required to generate this activity change should match those that generate the dopamine response. Finally, if the response to the CS contributes to the dopamine phasic activity, blocking the cellular plasticity in the candidate structure should be sufficient to decrease dopamine neuron responsiveness to the CS.

Here, we hypothesise that the superior colliculus (SC), a primary sensory area in the midbrain, is the brain area that identifies the salience of visual cues in classical conditioning and drives dopamine phasic activity. The superficial layers of the SC receive visual input from the retina and relay it to the deep layers, which have direct glutamatergic projections to midbrain dopamine neurons^9^. When dis-habituated pharmacologically, the visual input through this pathway can drive midbrain dopamine neurons at a latency similar to that observed in classical conditioning^10^. By using in vivo field potential recording, multi-unit recording, fast-scan cyclic voltammetry, and calcium imaging in anaesthetised and freely moving animals, we show that the deep layers of SC respond to a visual CS similarly to dopamine neurons but faster with a consistent short latency. We propose this response to be the main trigger of dopamine neuron burst activity following a visual CS in classical conditioning.

## Results

### SC responds to conditioned visual stimuli like dopamine neurons

**Fig. 1:**
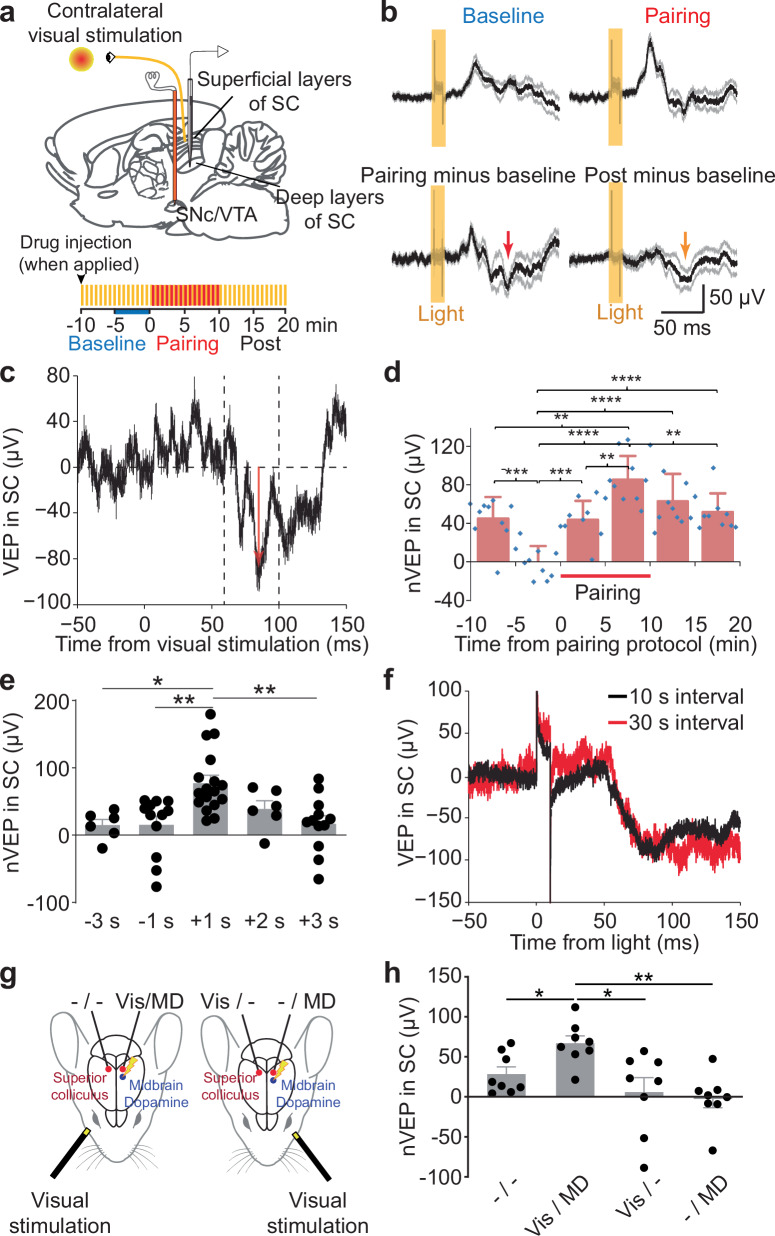
Potentiation of the negative VEP component (nVEP) in the deep layers of the SC in the rat. **a** Cartoon of the experimental setup on a sagittal rat brain section. Lower panel, a diagram of the experimental protocol with timing of light flashes alone (orange) and light flashes paired with SNc/VTA stimulation (red). Adapted from the Allen Rat Brain Atlas (Allen Institute for Brain Science; brain-map.org), CC BY 4.0. (https://creativecommons.org/licenses/by/4.0/). **b** Effect of a light flash on SC responses (S.E.M., grey traces) before and after SNc/VTA pairing (*N* = 9 animals). **c** Representative VEP from one animal showing the measured negative component (nVEP; arrowed at the trough of the VEP), where the trough happens at about 75 ms. **d** The mean maximum amplitude of the nVEP (*N* = 5 animals) between 50 and 100 ms after visual stimulation, plotted across time throughout the whole experiment (each blue dot represents the average of three consecutive nVEPs recorded during the protocol; histograms 5-min bins, inverted so negative is up; One-way ANOVA (*p* < 0.0001) with Tukey’s Multiple Comparison Test). **e** The nVEP is significantly potentiated only when light is applied 1 s before the SNc/VTA stimulation reward (One-way ANOVA, *p* = 0.0005, followed by Bonferroni’s Multiple Comparison Test). **f** nVEPs were induced regardless of whether the inter-pairing interval was 10 or 30 s within the same experiment. The trace shown is a representative recording from 5 repeats, all of which consistently demonstrated that both 10 and 30-s interval pairings induced nVEPs following light stimulation. **g** Experimental layout from top of the animal’s head: the VEPs in both SCs (red circles) were recorded simultaneously. Each eye (black semi-circles) was stimulated alternately, and midbrain dopamine neurons (MD) on the left (blue circles) were stimulated following each light flash. Image created using elements from SciDraw.io (CC-BY 4.0). (https://creativecommons.org/licenses/by/4.0/). Source (https://zenodo.org/records/3925903). **h** A significant nVEP component only developed when visual input and midbrain dopamine stimulation spatially converged (Vis/ MD group; *N* = 8 animals). Friedman test (repeated, nonparametric) with Dunns post hoc test. Data are means ± S.E.M. * *P* < 0.05, ** *P* < 0.01, *** *P* < 0.001, **** *P* < 0.0001. Source data are provided as a Source Data file.

For the SC to be the region where visual stimuli are associated with reward, it should develop a response to reward-associated visual stimuli that decreases during extinction. To test this, a flash of light into the eye was used as the CS, electrical stimulation of the substantia nigra pars compacta and ventral tegmental area (SNc/VTA) was used as the rewarding US^11,12^ and the associated visual evoked potential (VEP) was measured in the deep layers of the contralateral SC in anaesthetised rats (Fig. 1a and Supplementary Fig. S1). Stimulation of SNc/VTA was applied 1 s following the light flash to mimic a physiologically meaningful reward delay. The paired visual and SNc/VTA stimulation was applied for 60 pairings. Compared to the baseline, a negative deflection of the VEP (nVEP) developed (84.5 ± 7.8 µV; mean ± SEM) with a peak latency of ~75 ms (76.16 ± 4.02 ms; mean ± SD) (Fig. 1b, c). Notably, while the nVEP is likely driven by local spike activity in the deep layers of the SC, we also observed a positive phase of the VEP at ~30 ms, which is more likely attributable to spike activity in the superficial layers of the SC, whose neurons project to the deep layers.

Closer inspection of the VEPs throughout the experimental protocol (Fig. 1d; *N* = 5 animals) revealed that the nVEP gradually decreased during the baseline period, when light was applied alone, reflecting habituation of the visual response in the superior colliculus. Also, nVEP not only gradually potentiated when visual stimulation was paired with reward but also then decreased during extinction, i.e., when the visual stimulus was disassociated from the reward, over the 10 min following pairing. Also, consistent with previous reports^13^, response to the initial visual stimulation habituated quickly during baseline testing (−10 to 0 min). There was a trend for nVEP extinction to be slower compared with the initial habituation to baseline light flashes (Fig. 1d; *P* = 0.056, *t* = 2.67, df = 4). This might reflect a higher salience value encoded in the visual stimulus after it is paired with a reward compared with when it is novel^14,15^. Therefore, the amplitude and persistence of the nVEP in the deep SC increased with the number of rewarded visual stimulations and persisted beyond reward delivery, as described in reinforcement learning and the ‘shaping bonuses’ theory, respectively^16^.

### Critical temporal and spatial afferent convergence

We then tested whether the latency of the nVEP induced by classical conditioning was appropriate to trigger the dopamine response following a CS. It has been shown that local injection of bicuculline (BIC), a GABA_A_ receptor antagonist, into the SC in an anaesthetised animal disinhibits the SC, leading the deep layers of SC to respond to the visual stimulation with a nVEP and a response in midbrain dopamine neurons at a latency that is similar to classical conditioning^10,17^. Here we found that the nVEP induced by SNc/VTA pairing showed an onset latency similar to the nVEP induced by BIC ( ≈ 60 ms), although with smaller amplitude (80 µV pairing; 330 µV post-BIC) and earlier peak latency (80 and 100 ms respectively; Supplementary Fig. S2). Therefore, the timing of the nVEP generated in the SC is an excellent candidate for driving the dopamine response during classical conditioning. This also suggests that the mechanism underlying the pairing-induced potentiation of the nVEP may involve, in part, a reduction in inhibition within the collicular microcircuit.

Classical conditioning is optimal at particular inter-stimulus-intervals between CS and US^18^. To test if this is true for the potentiated nVEP, we applied SNc/VTA stimulation at various times before or after visual stimulation (Fig. 1e). The nVEP was significantly larger when SNc/VTA stimulation was applied 1 s after the light compared to 3 s before, 1 s before, or 3 s after. Pairing at 2 s induced an intermediate potentiation. The potentiated response following 1 s pairing was insensitive to pairing frequency, with a similar potentiated response resulting for inter-pairing intervals of 10 or 30 s (Fig. 1f). Therefore, nVEP potentiation can be induced through low pairing frequencies, with each pairing occurring within a behaviourally-relevant critical time window, consistent with behavioural observations in classical conditioning^18^.

The brain structure that develops the response to the CS should receive information from both the CS and US to identify their association. To test whether the potentiated nVEP in SC fits this requirement, we alternated the visual stimulus between the two eyes while activating the midbrain dopamine neurons on the left side only (Fig. 1g). In rats, more than 90% of visual input to the SC is contralateral^19^, and almost all projections from the SNc and VTA are restricted to the same hemisphere^20^. We found that only the nVEPs in the SC, where there was convergence of visual input (from contralateral side) and midbrain dopamine neuron activation (from the ipsilateral side) during pairing were potentiated (Fig. 1h). None of the other combinations of light flash and midbrain dopamine neuron stimulation elicited a comparable visual response. The −/− group, in which the recording site was not expected to receive either visual or dopamine input, showed a slightly higher response than the other two control groups, though still much smaller than the paired condition. Thus, potentiation of the nVEP required a spatial convergence within the same hemisphere of activation of the primary sensory area in the SC and midbrain dopamine neuron activation.

### The nVEPs represent local spike activity in deep layers of the SC

**Fig. 2:**
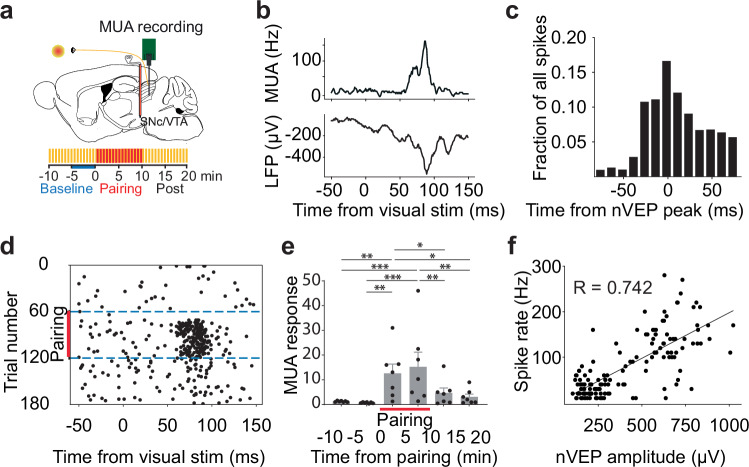
Amplitudes of nVEPs represent the number of action potentials generated from neurons in the deep layers of the SC in the mouse. **a** Cartoon of the experimental setup. Adapted from the Allen Mouse Brain Atlas (Allen Institute for Brain Science; brain-map.org), CC BY 4.0. (https://creativecommons.org/licenses/by/4.0/). **b** Example average multi-unit activity (MUA) trace during pairing and LFP trace (pairing minus baseline) in a representative mouse, showing the nVEP peak coinciding with the MUA peak. **c** Histogram of the fraction of spikes during pairing across all responsive channels (*n* = 2926 spikes in 7 channels, *N* = 4 animals) across times from the nVEP peak in each trial. **d** Example raster plot of the MUA response to light stimulus in a representative channel. **e** MUA response magnitude time course in all responsive channels (7 channels, *N* = 4 animals) as a fraction of the mean pre-pairing baseline response in each channel. (One-way ANOVA, *p* = 0.0408, followed by Fisher’s LSD post hoc test, data are Mean ± S.E.M. * *p* < 0.05, ** *p* < 0.001, *** *p* < 0.0001). **f** Scatter plot of nVEP amplitude versus MUA response spike rate for each trial in an example channel across a complete experiment. Source data are provided as a Source Data file.

We further confirmed that the nVEP in the deep layers of the SC represents local neuronal spike activity. The negative deflection of the local field potential has long been used as a proxy for excitatory input and local spike activity in brain areas such as the hippocampus^21^. To determine if the nVEP is correlated to local neuronal activity in the SC, we recorded spike activity and the nVEP simultaneously in the mouse SC using silicon probes (Fig. 2a).

First, we successfully replicated our findings regarding basal nVEPs in the mouse SC, where nVEPs with similar latency and comparable amplitude were observed when neutral visual stimuli were paired with electrical stimulation of the mouse SNc/VTA. Additionally, the local action potentials and nVEP matched well in latency and magnitude (Fig. 2b, c).

We then found that the firing of local action potentials developed similarly to the nVEP during pairing, increasing during pairing and reducing when electrical stimulation was removed (Fig. 2d, e). However, the habituation of spike activity was less prominent than that of the nVEP, which may reflect both the nonlinear relationship between spike activity and field potentials, as well as species differences in the encoding of naïve salient responses between mice and rats. Importantly, the number of action potentials recorded in the deep layers of the SC was highly correlated with the amplitude of the nVEP recorded simultaneously (Fig. 2f). These results suggest that the nVEP recorded in the deep layers of the SC is indeed associated with local spike activity.

### Rewarding midbrain dopamine stimulation also induces nVEP potentiation

**Fig. 3:**
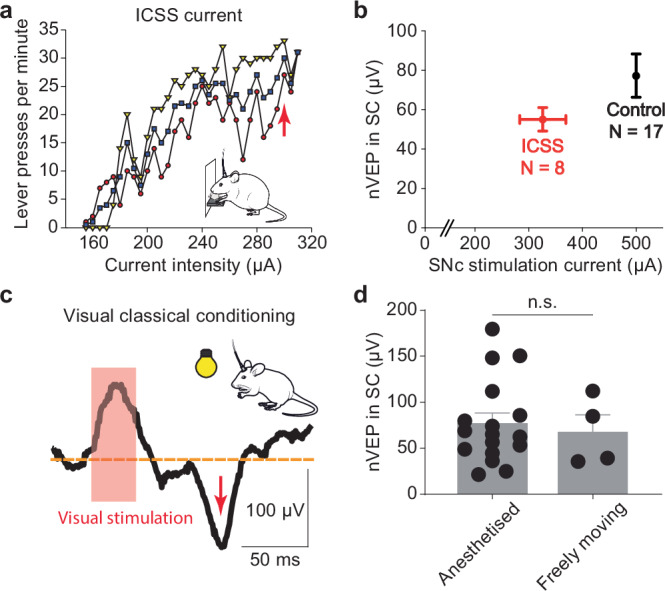
The nVEP component could be induced in both anaesthetised and freely moving rats, the latter receiving rewarding ICSS currents. **a** Lever-pressing rate for one rat in response to increments (red circles) and decrements (yellow diamonds) in SNc/VTA stimulus intensity. Arrow indicates the optimal current that maximises the average rate (blue squares). Animal image was created using elements from SciDraw.io (CC-BY 4.0). (https://creativecommons.org/licenses/by/4.0/). Source (https://zenodo.org/records/3925937). **b** nVEPs in anaesthetised trained rats induced using optimal ICSS currents and 10 ms visual stimuli were comparable to those obtained in naïve rats using a standard current of 500 µA (two-sided unpaired *t*-test, data are Mean ± S.E.M.). **c** An example VEP trace recorded from a freely moving rat (rat 1004), showing the nVEP component (arrow) following 1 s pairing between visual stimulation (30 ms; red bar) and ICSS-like stimulation. Animal image was created using elements from SciDraw.io (CC-BY 4.0). (https://creativecommons.org/licenses/by/4.0/). Source (https://zenodo.org/records/3925937). **d** nVEPs were of similar amplitude in both anaesthetised naïve rats and freely moving rats (two-sided unpaired *t*-test, data are Mean ± S.E.M.). Source data are provided as a Source Data file.

To determine whether potentiation of the SC response could be induced using known behaviorally rewarding stimulation, stimulating electrodes were implanted in the SNc/VTA in eight rats, and the animals were operantly conditioned using an intracranial self-stimulation (ICSS) protocol^12^. Animals were then anaesthetised, and the VEP was recorded using the same stimulus protocol, with the stimulus current that was identified for each animal to be behaviorally rewarding^12^ (Fig. 3a, b). The rewarding current potentiated the nVEP in the SC in ICSS rats by a magnitude similar to that observed in naïve rats (Fig. 3b).

We then tested whether the nVEP in the SC would be potentiated during classical conditioning in awake animals. We first determined the intensity of the rewarding electrical stimulation on SNc/VTA by using an operant ICSS protocol^12^. Then, freely moving rats were placed in a chamber for visual classical conditioning. A bright whole-field visual stimulus was paired with their ICSS reward currents and the nVEP was recorded in the SC in response to the visual stimulus (Fig. 3c). We found the same potentiated SC response in these freely moving animals (68.0 ± 18.5 µV, *N* = 4) as recorded in the anaesthetised rats (Fig. 3d). Moreover, following pairing, animals responded to the whole-field light flash with increased movement, i.e., running (Supplementary Fig. S3), consistent with others’ observations in classical conditioning^22^. This contrasted with the absence of such movements in response to the light during baseline or prolonged extinction (> 10 min), suggesting they had learnt through pairing the significance of the light flash as a predictor of SNc/VTA reward delivery. The change in the animal’s activity, calculated as the difference in speed of movements 0.5 s after compared to 0.5 s before the light flash, correlated with the amplitude of the potentiated VEP (*r* = 0.98, *P* < 0.05). In contrast, the change in movements in response to the midbrain dopamine stimulation did not correlate with the nVEP (*r* = 0.09, *P* = 0.91), indicating that potentiation of the nVEP was related to the conditioned change in response speed rather than the reward delivery (Supplementary Fig. S3). Therefore, potentiation of visual responses in the deep layers of the SC was induced in behaving animals and was associated with acquired responsiveness to a visual CS.

### Neuromodulator role in the potentiation of the SC neuronal response

**Fig. 4:**
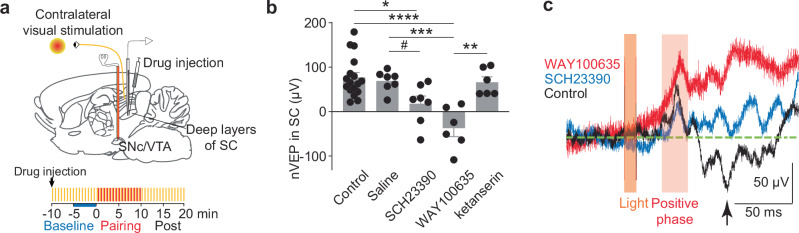
Potentiation of the nVEP component required dopamine D_1_ and serotonin 5-HT_1A_ but not 5-HT_2A_ receptor activation within SC. **a** Cartoon of the experimental setup on a sagittal rat brain section with indication of local drug injection into the deep layers of SC in anaesthetised rats. Adapted from the Allen Rat Brain Atlas (Allen Institute for Brain Science; brain-map.org), CC BY 4.0. (https://creativecommons.org/licenses/by/4.0/). **b** The nVEP in the deep layers of the SC was blocked by local injection of SCH23390 (D1 antagonist) and WAY100635 (5-HT1A antagonist) but not saline or ketanserin (5-HT2A antagonist). One-way ANOVA, *p* < 0.0001, followed by Bonferroni’s Multiple Comparison Test. # Two-sided paired *t*-test (*p* = 0.0434). Data are Mean ± S.E.M. **c** The control group showed an enhanced negative component (arrow), but not in SCH23390 and WAY100635 groups. Source data are provided as a Source Data file.

In order to investigate the cellular mechanisms underlying the potentiation of SC VEPs, antagonists to dopamine or serotonin (5HT) receptors were locally injected into the SC immediately before the commencement of the light flash baseline (Fig. 4a). Both the D_1_-like dopamine receptor antagonist SCH23390 (17.8 ± 17.3 µV) and the 5-HT_1A_ receptor antagonist WAY100635 (−36.8 ± 19.4 µV) significantly blocked the induction of the potentiated nVEP component compared to the control (uninjected) group (Fig. 4b, c). Local saline injection (69.1 ± 8.8 µV) did not affect the potentiation of the nVEP compared to the control (uninjected) group (77.2 ± 11.1 µV) (Supplementary Fig. S4), but was significantly different to the SCH23390 and WAY100635 groups. In contrast, the 5-HT_2A_ receptor antagonist ketanserin did not affect the induction of the nVEP component (66.3 ± 12.3 µV) when compared to either normal or saline controls (Fig. 4b and Supplementary Fig. S4). Collectively, these results suggest that potentiation of the nVEP in the deep layers of the SC is mediated by local dopamine acting on D_1_ receptors and serotonin acting on 5-HT_1A_ but not 5-HT_2A_ receptors. This provides functional evidence of dopamine and serotonin presence in the deep layers of the SC and their role in potentiation of visual responses.

### US and CS are associated in the deep layers of the SC

We next investigated whether the deep layers of the SC process the visual inputs to identify their salience, rather than simply relaying them. Of note, the positive deflection of the VEP (pVEP) recorded in the deep layers of the SC may reflect neuronal activity originating in the superficial layers (Supplementary Fig. S5). Interestingly, the pVEP also potentiated during the pairing protocol ( ≈ 30–40 ms latency; see Fig. 1c). However, when the nVEP was diminished by local injection of SCH23390, the enhancement of the pVEP persisted, similar to that observed under saline treatment in the same animals during CS-US pairing (Supplementary Fig. S4). This indicates that different cellular mechanisms are operating in superficial and deep layers of the SC. Taken together, our data suggest that the potentiation of the nVEP, representing the conditioned visual stimulus, arises specifically within the intermediate/deep layers of the SC.

### Potentiation of visual responses in SC drives striatal dopamine release

**Fig. 5:**
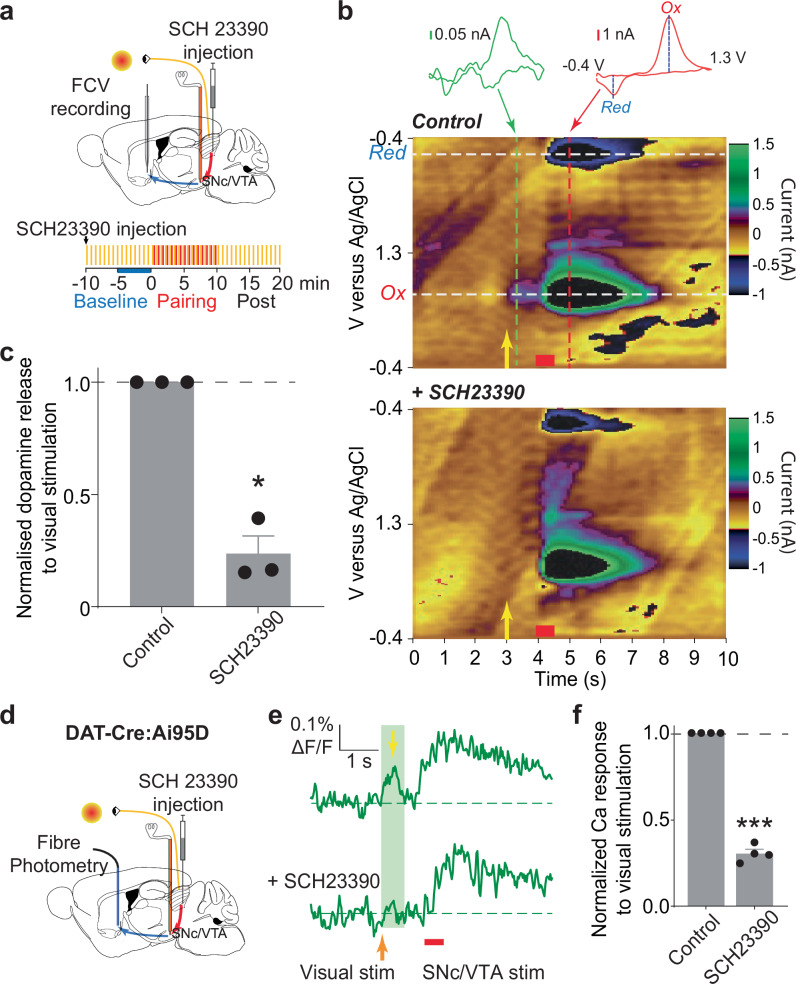
SC drives dopamine release in the mouse striatum through classical conditioning. **a** Striatal dopamine release via activation of the tectonigral (red arrow) to mesostriatal/mesoaccumbens (blue arrow) pathways was disrupted by local injection in the deep layers of SC of D1 antagonist, SCH23390, but not saline as control. Adapted from the Allen Mouse Brain Atlas (Allen Institute for Brain Science; brain-map.org), CC BY 4.0. (https://creativecommons.org/licenses/by/4.0/). **b** A representative colour plot of FCV recording during the pairing of visual (yellow arrow) and electrical stimulation (red bar) before (upper) and after (lower) SCH23390 injection. Black indicates saturated values in small scales (see Supplementary Fig. S5 for normal scale). Ox, oxidation potential of dopamine; Red, reduction potential of dopamine. The top inset shows voltammograms following visual (green) and electrical (red) stimulations. **c** SCH23390 blocks dopamine release following visual stimulation (via tectonigral to mesostriatal/mesoaccumbens pathway) but not electrical (mesostriatal/mesoaccumbens pathway alone) stimulation (two-sided one-sample *t*-test, *p = 0.0105, *N* = 3, data are Mean ± S.E.M.). Dopamine release following visual stimulation after SCH23390 administration was normalised to the control (saline) recordings performed in the same animals. **d** fibre photometry recording of GCaMP6f signal in NAcc. Adapted from the Allen Mouse Brain Atlas (Allen Institute for Brain Science; brain-map.org), CC BY 4.0. (https://creativecommons.org/licenses/by/4.0/). **e** Examples of Ca^2+^ imaging responses in dopamine axons in NAcc. The yellow arrow indicates visual response. **f** local injection of SCH23390 in SC blocked dopamine axon responses to visual stimulation in the NAcc (two-sided one-sample *t*-test, * *p* = 0.0012, *N* = 4, data are Mean ± S.E.M.). GCaMP6f activity in dopamine neurons following visual stimulation after SCH23390 administration was normalised to the control (saline) recordings performed in the same animals. Source data are provided as a Source Data file.

Finally, we tested whether conditioning of the nVEP in the SC is sufficient to drive midbrain dopamine neurons to release dopamine in target areas during visual classical conditioning. Using in vivo fast-scan cyclic voltammetry (FCV), we monitored extracellular dopamine in the ventral striatum of anaesthetised adult C57Bl6/J mice (Fig. 5a). To amplify dopamine signals, cocaine (20 mg/kg), a dopamine reuptake inhibitor, was injected intraperitoneally. The same pairing protocol that potentiated the nVEP in the SC induced a new striatal dopamine signal in response to the light, which had been absent at baseline (Fig. 5b). To confirm that potentiation within the SC was causing CS-evoked dopamine release, SCH23390 (which blocked the nVEP potentiation in the SC) was injected locally into the deep layers of the SC. We found that SCH23390 significantly attenuated dopamine release elicited by visual stimulation following pairing (23.7 ± 7.9% compared to control) but left intact dopamine release in response to electrical stimulation of the SNc/VTA during pairing (95.9 ± 13.1% compared to control; Fig. 5b, c and Supplementary Fig. S6).

Additionally, we measured dopamine axon activity in the NAcc by imaging calcium signals, which are more readily detectable than dopamine by FCV and do not require cocaine to amplify the signal (Fig. 5d). Fibre photometry recording of GCaMP6f in NAcc in DAT-Cre:Ai95D mice showed again that local application of SCH23390 in the deep layers of the SC significantly attenuated dopamine axon activity in the NAcc following visual CS (30.6 ± 2.0% compared to control; Fig. 5e, f).

Notably, the dopamine release and calcium signal in dopamine axons recorded in NAcc are most likely triggered by dopamine cell body activity. Cholinergic interneurons, as a possible alternative mechanism, are too slow to receive visual input and drive the observed dopamine release, as it takes more than 200 ms for visual stimulation to evoke burst activity in cholinergic interneurons that may promote axonal dopamine release^23^ or may even prevent^24^ dopamine axon activity. Therefore, dopamine-dependent potentiation of the nVEP in the deep layers of the SC is able to drive dopamine release into the striatum following CS.

## Discussion

We have demonstrated that the deep layers of the SC identify the salience of the visual CS, under conditions where the unconditional salience is imposed via midbrain stimulation. The potentiated response developed in the deep layers of SC can drive the midbrain dopamine neurons to release dopamine in the striatum at the latency of visual classical conditioning. Many brain areas that have inputs to the midbrain dopamine neurons have been discovered to carry information about classical conditioning^25^. However, the brain structure where the response to the CS originates is still unidentified. Our study confirms that the deep layers of the SC form an association between a CS and US during visual classical conditioning and drive downstream dopamine responses.

**Fig. 6:**
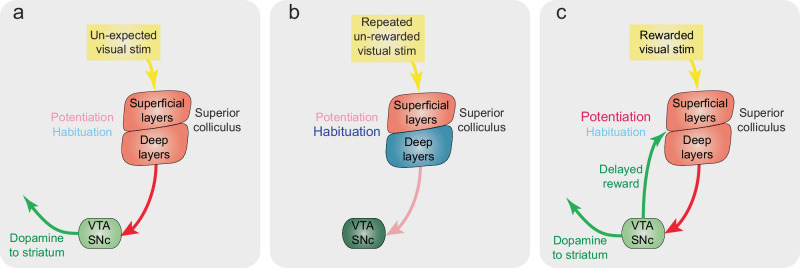
A model for how the deep layers of SC integrate information of US and CS and drive striatal dopamine release during stages of visual classical conditioning. **a** A salient un-expected visual stimulus drives the deep layers of the SC to activate dopamine neurons in SNc/VTA. **b** When the visual stimulus loses its salience, the deep layers of the SC habituate to the stimulus. **c** The delayed dopamine signal elicited by the US potentiates the visual response and the SC starts to drive dopamine release into the striatum following visual stimulation (CS). The balance of habituation and potentiation, therefore, controls visual responsiveness in the SC. The figure was created using Adobe Illustrator (Adobe Inc.).

Understanding how activation of the dopamine neurons following a US modulates their own firing response to a CS alone is critical for understanding the processes of classical conditioning. Our results suggest that the dopamine signal that accompanies a US potentiates the neuronal response in SC elicited by a CS such that a CS alone can drive the dopamine neurons to release dopamine in target areas. By introducing this feed-forward loop into the circuit, our model (Fig. 6) provides a possible explanation of the modulatory effect of dopamine in classical conditioning, at least for visual stimuli.

In the current study, we applied electrical stimulation to electrodes in the dopamine cell layer (Supplementary Fig. S6) and used an electrically activated dopamine signal as the US to precisely control the interval of CS and US. Since there was no requirement for any movement by the animals to receive the reward, this paradigm was suitable for study in both anaesthetised and freely moving animals. We showed that the nVEP in the SC can be potentiated when a visual stimulus is followed by a reward 1 s later, which is consistent with action discovery paradigms in humans and animals^26,27^. In addition, as demonstrated, applying rewarding electrical stimulation before the visual stimulation did not evoke an nVEP in the deep layers of the SC, further supporting our conclusion that the SC responds to the CS-US association only when the US is applied at behaviourally meaningful intervals. However, it does not imply that the SC can only form conditioned associations when rewards are delivered within a critical 1 to 2 s delay. The 1 to 2 s optimal reward delay observed in our experiment might be specific for using SNc/VTA stimulation as a reward, which only paired with the visual stimuli 60 times. Also, many of our results were acquired with anaesthetised animals. It is possible that in awake behaving animals, longer reward delays may be bridged by behaviorally relevant inputs to SC from cortex. Moreover, it is possible that longer reward delays (> 2 s) could potentiate the nVEP after more pairings^2,28^ than the 60 pairings we used here.

We also found that a D_1_ receptor antagonist delivered to the SC blocked the development of the nVEP. In addition to the dopamine neuron projection from the midbrain to mid and deep layers of SC^29^, the SC receives dopamine innervation from A13 zona incerta^30^. In addition, all layers of the SC receive massive serotonergic projections from the raphe nuclei^31,32^, and express dense 5-HT_1A_ and 5-HT_2_ receptors^33^. Here we show that 5-HT_1A_ but not 5-HT_2A_ receptors are involved in forming an association between CS and US in deep layers of SC. While blocking 5-HT_1A_ receptors reduces the excitatory effect of optic tract stimulation on SC neurons^33^, and ketanserin (a 5-HT_2_ antagonist with primary action at 5-HT_2A_ receptors) attenuates the excitatory effects of 5-HT on SC neurons^34^, our results suggest that serotonin is involved in associating visual SC and US by modulating the excitatory effect of the optic input onto SC neurons. In addition, the 5-HT_1A_ antagonist could also exert effects beyond the nVEP itself, as suggested by the prolonged responses. This raises the possibility that the drug may act through a broader inhibitory or modulatory influence on local network activity within the superior colliculus, rather than exclusively targeting the nVEP.

Dopamine neurons can behave in a similar pattern when responding to a CS during classical conditioning^8^, making it possible that they are driven by a common input. Neurons in many brain areas sending input to dopamine neurons have been shown to encode the saliency of sensory cues in classical conditioning^35,36^. While dopamine neurons increase their firing rate in response to a CS, this response is likely caused by excitatory input to dopamine neurons. Here, our data suggest that the deep layers of the SC drive dopamine neurons to respond to a CS in classical conditioning via their excitatory glutamatergic input. This conclusion supports a recent finding that glutamatergic input may drive dopamine phasic activity in response to a CS^37^.

Our research not only provides insights into the functioning of the SC but also has practical implications. The potentiated neuronal response in the SC not only drives the dopamine neurons during classical conditioning, but also provides a physiologically meaningful measure of the “eligibility” trace. This trace, a short-lasting neural effect induced in the SC by the light flash, is operated on by reward within a critical timing interval (less than 2 s). By measuring neuronal activity, we will be able to study the eligibility trace for visual stimuli in future, where the nVEP within the SC may provide a temporally precise signal that plays a critical role in the eligibility trace. This method could be extended to characterise the eligibility trace for other sensory modalities and using other behavioural paradigms, potentially leading to new approaches in the field of neuroscience and psychology.

We note that our experimental paradigm is unable to distinguish the different components of the dopamine response to the CS. It has been demonstrated that dopamine neurons can respond to a CS with two distinct components when the CS is sufficiently complex, such as when it includes information about the probability of receiving a reward. While the first large component encodes the novelty of the CS, the smaller second component can encode the reward probability^38^. In our experiment, a simple light flash was used as the CS, and it indicates a 100% probability of reward during the pairing of CS and US. Therefore, the potentiated neuronal response in the deep layers of the SC we recorded may only contribute to dopamine response component that encodes the novelty of the CS.

In summary, we propose that early sensory processing in the deep layers of the SC encodes the value of visual stimuli via the process of classical conditioning. The visual response in this structure represents a dynamic balance of potentiation, elicited by a dopamine/serotonin signal in the SC driven by the US (directly or indirectly), and the properties of habituation, which by default attenuates the response to unreinforced stimuli (Fig. 6). This mechanism of sensory conditioning would ensure that the system remains dynamically responsive to biologically significant sensory events. Importantly, it offers a potential explanation of not only the extensively reported sensory response characteristics of dopamine neurons^15,39^, but also the enhanced ability of biologically salient stimuli to attract attention^40–42^.

## Methods

All experimental procedures with rats were approved by the University of Otago’s Animal Ethics Committee in accordance with the NZ Animal Welfare Act 1999. All procedures with mice in FCV and multi-unit experiments were performed in accordance with the UK Animals (Scientific Procedures) Act 1986 (Amended 2012) with ethical approval from the University of Oxford and under the authority of a Project Licence granted by the UK Home Office.

### Surgery for recording with naïve rats

A total of 68 male Long-Evans rats weighing 350–390 g were anaesthetised with an initial dose of urethane (1.6–2.0 g/kg, Sigma-Aldrich, intraperitoneal; i.p.) and an additional dose 15 min later (0.6 g/kg, i.p.). Before surgery, a local anaesthetic (bupivacaine, 0.5%) was injected into the scalp. Supplementary doses, 0.2 ml, of urethane (0.6 g/kg) were given through an i.p. cannula during experiments to maintain the anaesthesia level. Core temperature was maintained at 35–36 °C during the experiment using a homoeothermic blanket and rectal probe (TR-100, Fine Science Tools). A concentric stimulating electrode (Rhodes NEW-100 × 10 mm, USA) was implanted through a skull hole into the SNc/VTA in the left hemisphere (AP −4.8 mm and ML 1.5 mm to Bregma, at a depth of 7.7–7.8 mm from the brain surface). Electrode positions were verified in cresyl violet stained sagittal sections (Supplementary Fig. S7).

### Surgery for recording multi-unit activity with mice

C57Bl/6 mice were anaesthetised with an i.p. injection of urethane (1.5 g/kg). A local anaesthetic (0.5% Bupivacaine) was injected into the scalp before surgery. Core temperature was maintained at 36 °C during the experiment using a homoeothermic blanket and rectal probe (Harvard Apparatus). Eye lubricant was applied at the beginning of the surgery, and the light source was placed ca. 1 cm from the contralateral eye. The stimulation electrode was placed in the SNc/VTA (AP −3.1, ML 0.8, DV −4.2), and the recording electrode was placed in the lower SC (AP −4.0, ML 0.8, DV −2.1). Silicon 15-channel electrodes (Cambridge Neurotech) were used, with a triangular electrode arrangement at the tip spanning 150 µm vertically. A reference wire was introduced into the contralateral cerebellum.

### Behavioral methods

Seven male Long-Evans rats (350 ± 390 g) were implanted for ICSS. Rats were anaesthetised with ketamine (60 mg/kg, i.p., Phoenix Pharm Distributors Ltd, NZ) and domitor (0.5 mg/kg, i.p., Orion Pharma Pfizer, Finland) and placed in a stereotaxic frame. Prophylactic antibiotic was administered subcutaneously. A bipolar stimulating electrode (PlasticsOne MS303/2, USA) was positioned at the level of the SNc/VTA as in naïve rats. At least three days were allowed for post-operative recovery. Rats were trained to perform ICSS in a behavioural chamber with a lever that, when pressed, immediately triggered delivery of a single stimulus train (100 Hz, 0.5 ms biphasic pulses, 500 ms train duration) to the SNc/VTA electrode. The response rate versus current intensity data was collected daily. The training ceased when the optimal current was stable to within 10% for three consecutive sessions (Fig. 4a).

In behavioural conditioning experiments, the animals were placed in the recording chamber and allowed to settle for 15 min in the dark. A program written in MedPC (Med Associates, USA) then began to deliver a short activation (30 ms) of five stimulus lights around the chamber, ganged to achieve whole-field visual stimulation, at an interval of 10 s. After a 10-min baseline period of light flashes alone, each light presentation was followed 1 s later by the delivery of a single stimulus train to the animal’s SNc/VTA at their optimal current. The LFP in the deep layers of the SC was recorded for offline analysis. The behavioral response to the light was video recorded throughout the experiment and analysed offline using TopScan behavioral analysis software. The average speed of the animal’s movements in 0.5 s bins around each light flash was measured and exported to Excel files. The difference in speed 0.5 s before and after each light flash was calculated during the pairing protocol and during the baseline period where the light flash was delivered alone prior to pairing. The difference in speed between 0.5 s after the SNc/VTA stimulation and the period prior to the light flash during pairing was also calculated for comparison.

### Local field potential recording

The LFP in the superior colliculus was recorded using a glass electrode (1–2 MΩ) pulled from calibrated glass capillaries (volume 5 µl, diameter 1.0 mm; Modulohm I/S, Denmark) filled with 0.9% NaCl solution. The recording electrode was implanted in the SC (Supplementary Fig. S7) with a micromanipulator (IVM, Scientifica, UK) at the position of AP −6.5 mm and ML 1.5 mm, or −1.5 mm when recorded from both hemispheres, to Bregma, at a depth of 4.0–4.1 mm from the brain surface. The coordinates were chosen to maximise the possibility of recording the LFP from the midpoint of the deep layers of the SC in the mediolateral plane, so the LFP did not only reflect the properties of medial or lateral SC^43^. A Teflon-coated tungsten wire was immersed in the NaCl solution and connected via a headstage (NL100 Neurolog) to a preamp (NL104), an amplifier (NL106) and a filter (NL125). The animal was grounded by a silver wire, which was introduced into the connective tissue underneath the animal’s back skin. Both signals were amplified and band-pass filtered (0.1–10,000 Hz). All waveform data were digitised at 50 kHz by 1401 Micro 2 (CED), displayed with Spike2 software (CED) and stored to disk. Behaving animal recordings were performed using a chronically implanted stainless steel wire and reference electrode screwed on the skull on the other side above the SC connected to a locally constructed battery-powered FET headstage, run with the stimulator wires to a commutator (PlasticsOne, USA) and then to a preamplifier and Neurolog recording system.

### Multiunit activity recording

Recordings with the Silicon 15-channel electrodes (Cambridge Neurotech) electrode were acquired using Intan Technologies data acquisition software (RHX) at 30 kHz, and the unfiltered data was stored to disk. Data analysis was done using custom-written MATLAB and Python scripts. Spikes were extracted from the unfiltered data using the Kilosort v2.0 software. All spikes on each channel were then pooled to represent the multi-unit activity. The multi-unit activity (MUA) response magnitude was calculated as the difference between the spike rate 50–100 ms after light stimulation and the baseline spike rate (from 1 s before light stimulation).

### Drugs

Drugs were injected through the glass recording electrode in the SC. The pipette was held by a pipette holder that was attached to polyethylene tubing with a 10 ml syringe as a pressure injector. Each drug, including BIC (0.01% in 250 nl 0.9% saline), SCH23390 (2 µg in 250 nl saline), WAY100635 (2 µg in 250 nl saline), ketanserin (2.5 µg in 500 nl saline), or saline (250 nl), was injected into the SC at a rate of 400 nl/min. All drugs were sourced from Sigma-Aldrich.

### Light and electrical stimulation

Light stimuli (10 ms duration, 0.1–0.033 Hz) were delivered by a white LED (1500 mcd) that was placed 1–2 cm directly in front of the right eye of the animal. The left eye was covered. In eight experiments, stimuli were delivered to both eyes alternately with the non-stimulated right eye covered. In the experiments with naive rats, SNc/VTA stimulation (0.5 ms diphasic pulses, 500 ms train duration; no damage noted in any experimental brain sections) was delivered at 100 Hz at a current of 500 µA. In the experiments with ICSS rats, SNc/VTA stimulation (100 Hz, 0.5 ms biphasic pulses, 500 ms train duration) was delivered using the optimal current acquired during training. Stimulation was controlled by locally developed software “StimulatorControl” (SCL Limited, New Zealand) on a Windows-compatible PC computer.

### Analysis of electrophysiological recording data

Data were analysed using Spike2 v6.09 and MATLAB 2012a, including customised MATLAB scripts. The average LFP was generated with a built-in function of Spike2. The onset time of visual stimulation was used as a trigger to create the average of the LFP over a 5-min recording (30 trials). The 50 ms period prior to the visual stimulation was defined as the baseline of the trace, and its average value was defined as 0 mV. The amplitude of the negative component of VEP (nVEP) was defined as the minimum value of the LFP during the period of 60–100 ms following visual stimulation (Fig. 1b). To minimise the influence of the recording noise, the amplitude was read as the average of eleven data points around the minimum value of the LFP.

Because the nVEP showed its minimum value at the last 5 min of baseline recording and its maximum value during the last 5 min of pairing, the difference of the average LFP between these two periods was defined as the amplitude of the nVEP response change (Fig. 1).

### In vivo fast-scan cyclic voltammetry (FCV)

A total of 16 adult (10–12 weeks) male C57Bl6/J mice were used for the in vivo FCV experiments. Mice were anaesthetised with urethane (1.5–1.8 g/kg), and surgical anaesthesia was maintained with supplementary isoflurane (1% w/o). Body temperature was maintained at 36–37 °C using a homoeothermic heating blanket. Corneal dehydration was prevented with eye ointment (Lacri-lube). After induction, the mouse was secured in a stereotaxic frame, the scalp was shaved and cleaned with dilute hibiscrub and 70% alcohol. A local anaesthetic, bupivacaine, was injected s.c. into the incision site. After the skull was exposed, holes were drilled for the Ag/AgCl reference electrode (AP: −2.0 mm, ML: 0.5 mm).

Extracellular DA concentration was monitored in the ventral striatum using FCV with 7 μm diameter carbon fibre microelectrodes (CFMs; tip length 50–100 μm). The scanning voltage was a triangular waveform (−0.4 to +1.3 V range vs Ag/AgCl) at a scan rate of 400 V/s and sampling frequency of 10 Hz, which was produced from a Tarheel system (USA). The data was acquired and analysed offline with custom-written Matlab (R2013b) scripts.

### In vivo fiber photometry

Round pieces of skull overlying the left hemisphere were removed to allow access to the DLS (AP +1.0 mm, ML 1.6 mm, DV 2.2 mm to Bregma) and SNc (AP −3.1 mm, ML 0.8 mm, DV 4.3 mm to Bregma). The injection and recording array, consisting of a glass pipette and a 200 µm diameter fibre, was positioned in the DLS. GCaMP6f expressed in DA axons in DAT-Cre:Ai95D mice was activated with 480 nm light (76 µW), and the intensity of GCaMP6f emission was sampled at 40 Hz with Neurophotometrics (FP3001).

For midbrain electrical stimulation, 0.5 mA current (500 µs) was delivered through a bipolar stimulating electrode (0.005 inch, MS303/3-A/SPC, P1 Technologies) at 0.1 Hz. The stimulating electrode tips were separated by ~500 µm.

### SNc/VTA stimulation and drug injection during in vivo FCV

A bipolar stainless steel stimulating electrode and a drug injection pipette pulled from a calibrated glass capillary (volume 5 µl, diameter 1.0 mm; Modulohm I/S, Denmark) were implanted in the SNc/VTA (AP −3.5 mm, ML −0.35 mm, DV 4.3–4.7 from brain surface) and SC (AP −3.5 mm, ML −1.0 mm, DV 1.8–2.2 mm from brain surface), respectively. SNc/VTA stimulation (0.5 ms biphasic pulses, 1200 ms train duration) was delivered at 50 Hz with the current at 500 µA. Note the stimulation frequency was chosen to avoid the clash of stimulation artefacts with FCV recording sweeps.

### Experiment protocol

The LFP recording in the SC was similar for all anaesthetized animal experiments:

1. Baseline recording: After the surgery, the rat was covered and kept within the dark for at least 10 min, so the animal adapted to the dark environment. Once the LFP stabilized, i.e., the LFP showed slow synchronized oscillation, the visual stimulation was applied to the right eye of the animal at 0.1 Hz for 10 min.
2. Pairing protocol: The pairing of visual stimulation and SNc/VTA stimulation was applied at 0.1 Hz for 10 min.
3. Post-pairing protocol: The visual stimulation was applied at 0.1 Hz for 10 min.

Drug injection: Drugs were injected directly before the baseline recording.

For each animal, at most two experiments were applied. There was a 30 min break between the experiments. During the break, no stimulation was applied to the animal.

### Histology methods

When the recording experiment was finished, the animal was perfused with a 0.1 M phosphate buffer solution (PBS) containing 4% paraformaldehyde. The brain was then removed and kept in fixative solution at 4 °C. Brains were sliced in 60 µm sections, which were then stained with a 0.03% cresyl-violet solution, and the correct position of the tracks was confirmed under the light microscope.

### Power calculations

To assess whether our sample sizes were sufficient to detect the reported effects, we performed post-hoc power analyses based on the observed effect sizes and variability in our data. For VEP measurements, the mean effect size of potentiation was approximately *d* = 1.2–1.5 (Cohen’s *d*), with a standard deviation of ~15–20 µV across animals. For these experiments, sample sizes of *N* = 4–7 animals provided statistical power of >80% to detect the observed effects at *α* = 0.05. For calcium imaging experiments, the mean change in signal amplitude was ~0.1–0.15 ΔF/F with within-animal variance of 0.02–0.04. Power analyses indicated that *N* = 4 animals were sufficient to achieve >80% power to detect these effects. For fast-scan cyclic voltammetry (FCV) recordings, the observed dopamine transients had large effect sizes (*d* = 1.0–1.4) and were highly reproducible across recording sites, with *N* = *3* animals yielding >75% power at *α* = 0.05.

Where possible, we used within-animal comparisons (e.g., baseline vs. post-pairing, or control vs. drug injection in the same animal), which substantially increases sensitivity by reducing between-subject variability. While we acknowledge that some *N* values are modest, these analyses indicate that the statistical power of our experiments was acceptable for the observed effect sizes. In addition, the convergence of findings across multiple methodologies (VEPs, MUA, calcium imaging, and FCV) provides further confidence in the robustness and reproducibility of our conclusions.

### Statistics

Analyses of the nVEP were carried out using one-way ANOVAs with Bonferroni’s Multiple Comparison post hoc test, Friedman tests (repeated, nonparametric) with Dunns post hoc test, or Paired *t*-tests. Analyses of dopamine release in the striatum were carried out using a one-sample *t*-test. Statistical analyses were performed using Prism5. Data are mean ± S.E.M. unless stated. Significance levels are indicated as follows: * *P* < 0.05; ** *P* < 0.01; *** *P* < 0.001; **** *P* < 0.0001.

### Reporting summary

Further information on research design is available in the Nature Portfolio Reporting Summary linked to this article.

## Supplementary information

Supplementary Information

Reporting Summary

Transparent Peer Review file

## Source data

Source Data

## Data Availability

Null Source data are provided with this paper.

## Code availability

The code used in this study is available from GitHub (https://github.com/Yanfeng-Zhang/SC-gates-visual-classcial-conditioning).
