# Supplementary material for: The superior colliculus gates dopamine responses to conditioned stimuli in visual classical conditioning
Source: Nat Commun. 2026 Apr 29;17:5885. doi: 10.1038/s41467-026-72167-4 (PMC13338361; doi:10.1038/s41467-026-72167-4)
Supplement: Supplementary file 1 — Supplementary Information [file 41467_2026_72167_MOESM1_ESM.pdf]

Supplementary figures for

The superior colliculus gates dopamine responses to conditioned stimuli in visual classical conditioning

Yan-Feng Zhang[1,2,3*], Jean-Philippe Dufour[3], Peter Zatka-Haas[3], Peter Redgrave[4], Melony J Black[1], Armin Lak[3], Ed Mann[2], Stephanie J Cragg[2], Wickliffe C Abraham[4], John NJ Reynolds[1*].

Correspondence to: Yan-Feng Zhang: y.f.zhang@exeter.ac.uk
John NJ Reynolds: john.reynolds@otago.ac.nz

This file includes:

Supplementary Figures 1 to 7

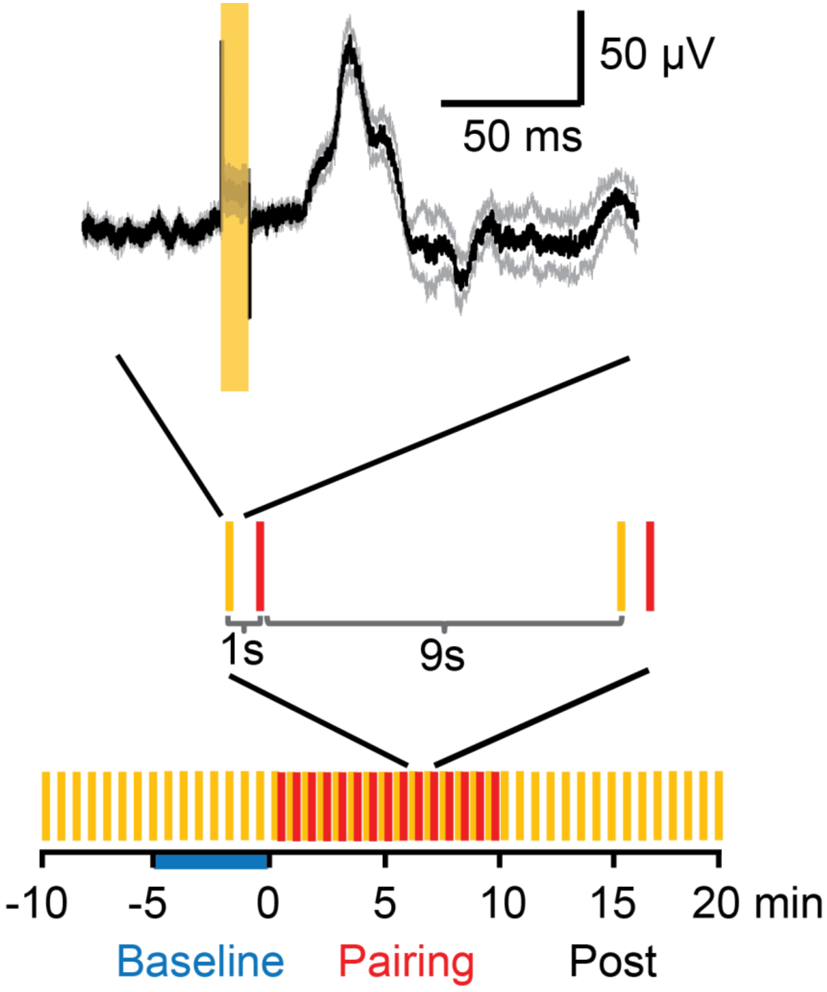

Supplementary Fig. 1. A diagram of the typical experimental protocol. Visual stimulation (orange) was applied every 10 s. To potentiate the short latency (60 – 100 ms) visual response in the deep layer of the SC (black and grey line, upper panel), electrical SNc/VTA stimulation (red) was applied 1 s after the visual stimulation, 60 times. Variations of these timing relationships were tested in some experiments.

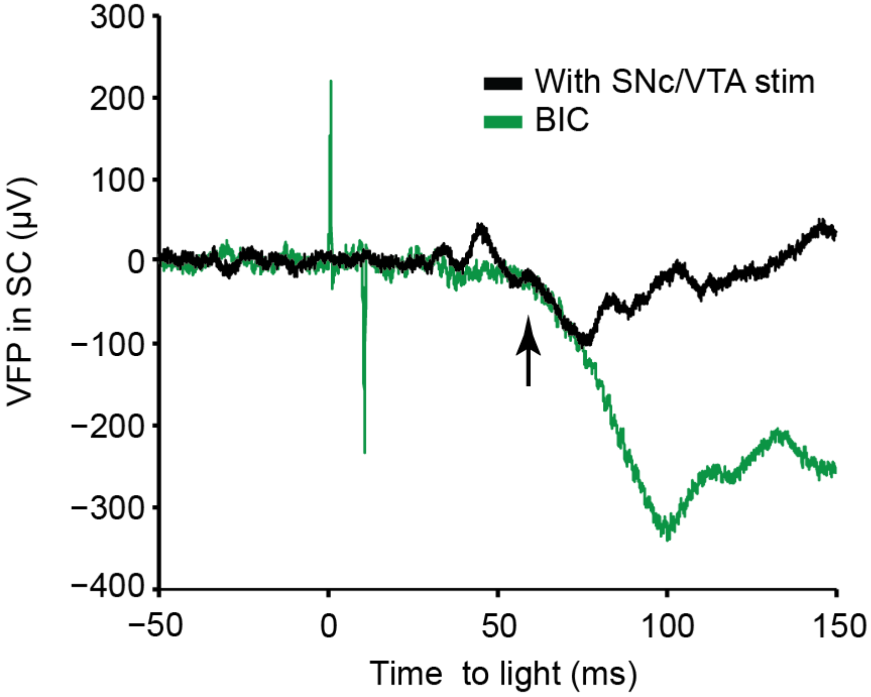

Supplementary Fig. 2. The VEP induced by visual classical conditioning shares similarities with the VEP induced by local injection of BIC in one rat. The potentiated nVEP component induced by pairing with SNc/VTA stimulation (black; pairing minus baseline trace) occurs at a similar onset latency (arrow) but earlier peak latency to the nVEP under the disinhibitory effect of BIC (green).

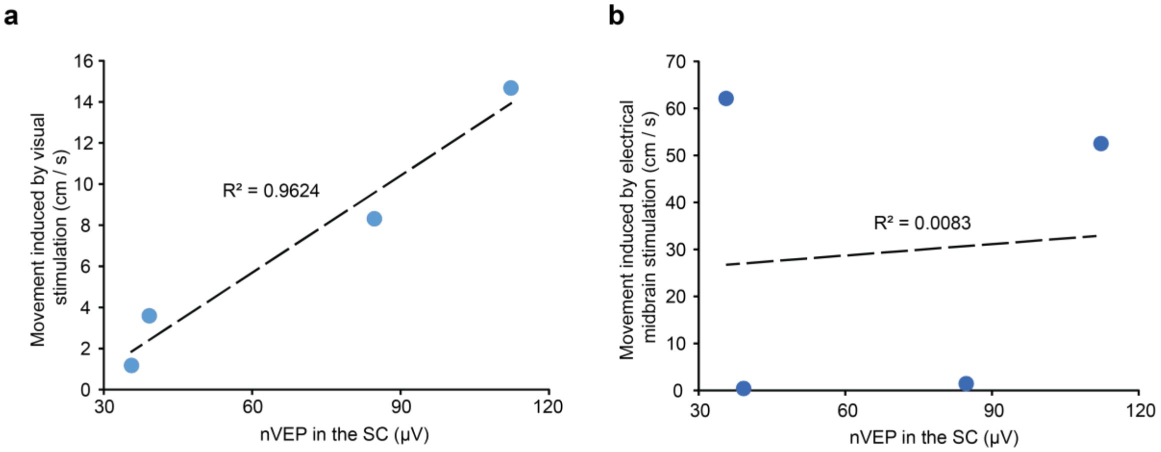

Supplementary Fig. 3. The development of nVEPs in the deep layers of SC correlated with the learning of the salience of the CS. a, The difference in the speed of movements in response to the light between the pairing protocol and the pre-pairing baseline period was positively correlated with the pairing minus baseline amplitude of the nVEP in the deep layers of SC. **b**, The difference in movement speed in response to the electrical stimulation was not correlated to the nVEP. Thus the change in the nVEP through pairing was related to the learning of the salience of the visual stimulus (CS; as indicated in a) and not to a general behavioral activation induced by the electrical stimulation (US).

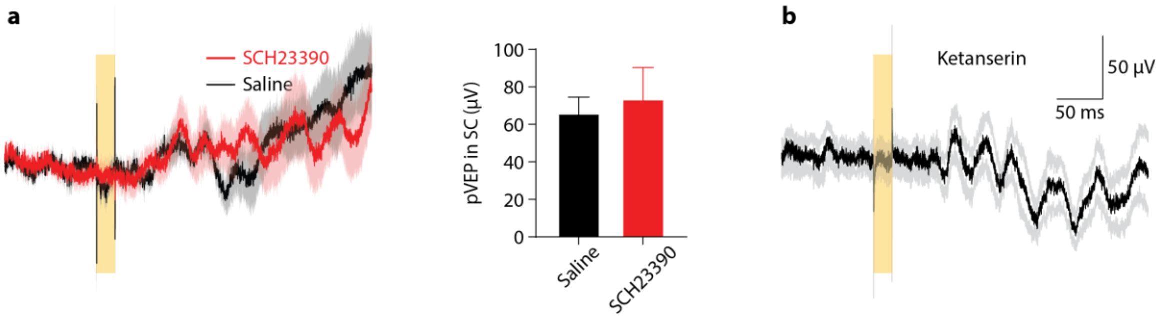

Supplementary Fig. 4. Local injection of saline or ketanserin did not abolish the VEP evoked by reward-predicting light stimulation. a, The pVEP (30 - 50 ms after visual stimulation) and nVEP (50 - 90 ms after visual stimulation) in the superior colliculus persisted following local injection of saline, whereas local injection of SCH23390 abolished the nVEP (N = 7 animals). b, The nVEP persisted following local injection of ketanserin (N = 6 animals). Traces represent mean ± s.e.m. (shaded or grey traces). Source data are provided as a Source Data file.

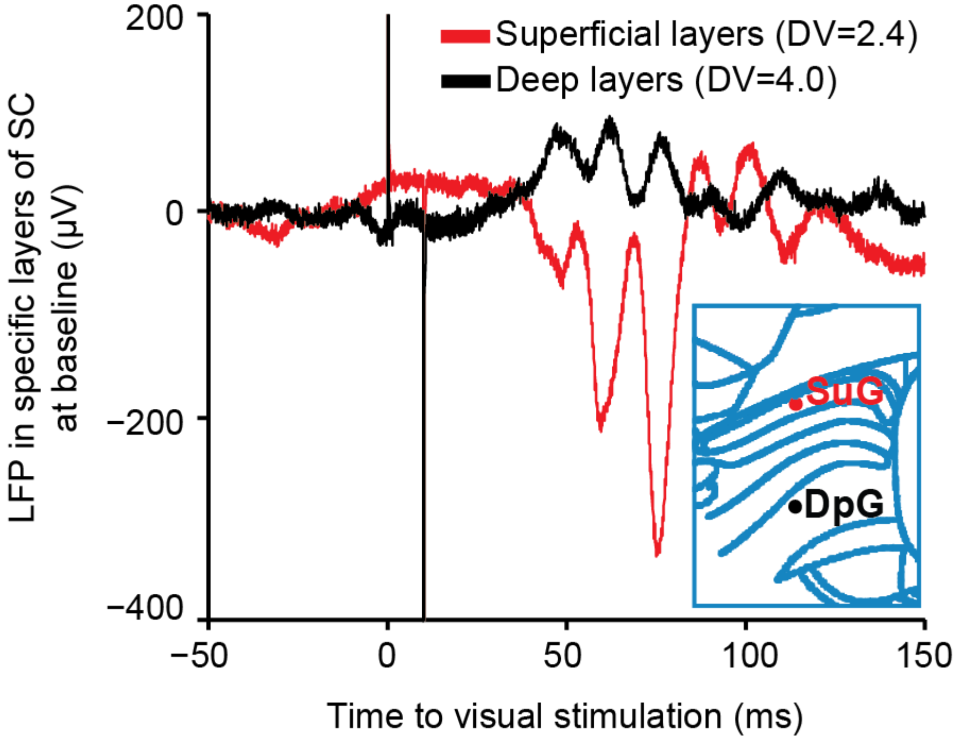

Supplementary Fig. 5. The representative (of 5 repeats) mean VEP in the deep layers (black) and the superficial layer (red) of the SC, recorded in one rat using the same electrode, before pairing. The positive component of the VEP in the deep layers is aligned in time with the negative component of the VEP in the superficial layers, indicating that pVEP in the deep layers may represent the current sink for the nVEP in superficial layers of the SC. SuG: superficial grey layer of the SC, DpG: deep grey layer of the SC.

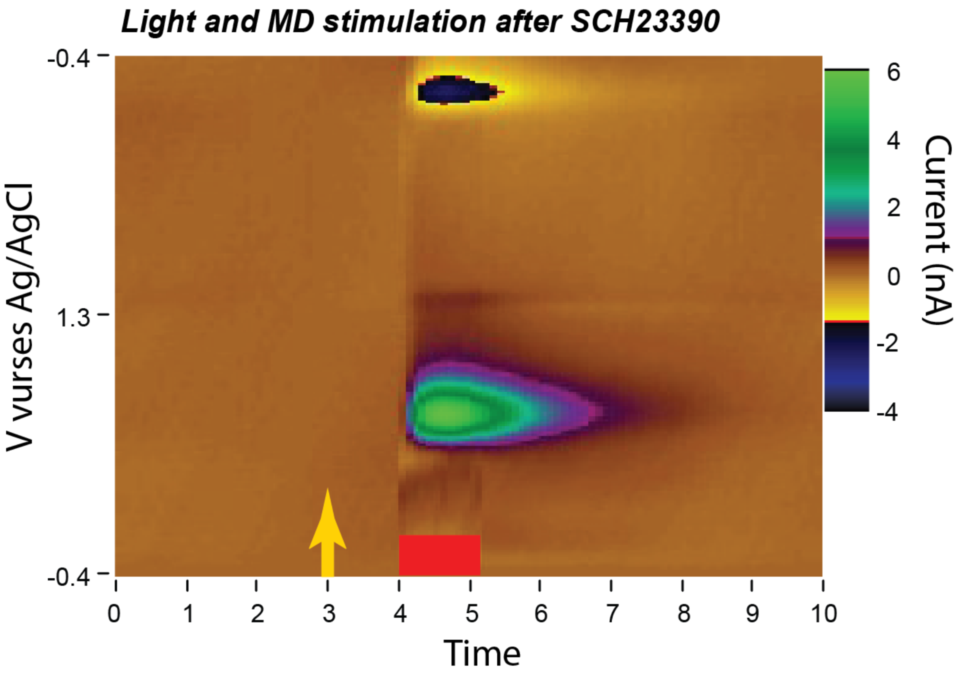

Supplementary Fig. 6. Dopamine release in response to the electrical stimulation during the pairing protocol on a normal scale. Local injection of SCH23390 blocks the dopamine release following visual stimulation, as in Fig. 5b. The scale is set to usual values to correctly show the dopamine release following midbrain dopamine (MD) electrical stimulation (red bar).

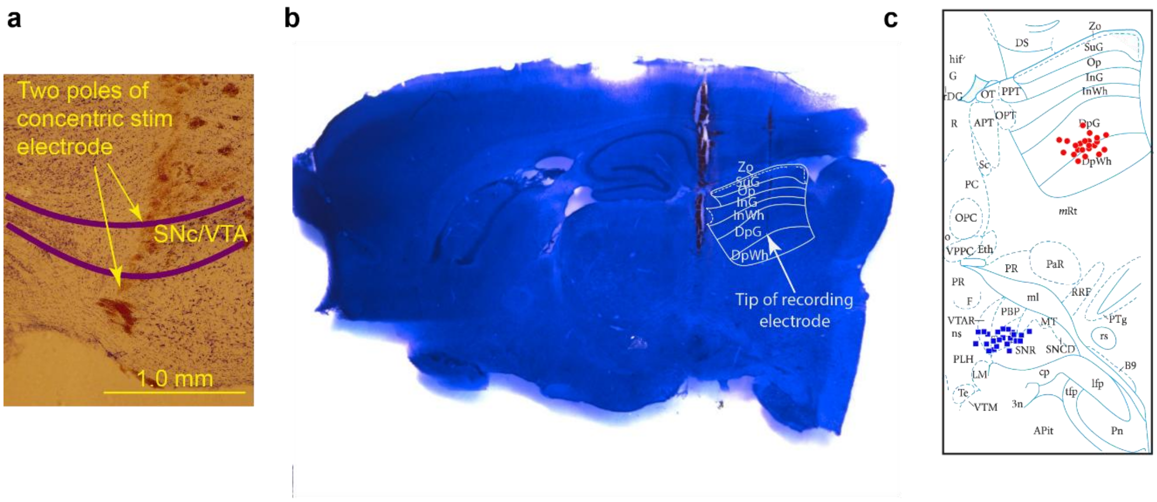

Supplementary Fig. 7. Example histological sections showing stimulating and recording electrode locations. a, The concentric stimulating electrode positioned to activate SNc/VTA. **b**, the tip of the recording electrode is positioned in the middle of the deep layers of the SC. Part of the stimulating electrode can also be seen more anteriorly in this sagittal section, in its path to the midbrain. DpG: deep grey layer, DpWh deep white layer of the SC. **c**, Approximate midpoint of the stimulating electrodes (blue squares, sagittal section at mediolateral +1.5 mm) and the position of recording electrode tips (red circles).
