# Supplementary material for: The superior colliculus gates dopamine responses to conditioned stimuli in visual classical conditioning
Source: Nat Commun. 2026 Apr 29;17:5885. doi: 10.1038/s41467-026-72167-4 (PMC13338361; doi:10.1038/s41467-026-72167-4)
Supplement: Supplementary file 3 — Transparent Peer Review file [file 41467_2026_72167_MOESM3_ESM.pdf]

nature portfolio

Peer Review File

The superior colliculus gates dopamine responses to conditioned stimuli in visual classical conditioning

Corresponding Author: Dr Yanfeng Zhang

This file contains all reviewer reports in order by version, followed by all author rebuttals in order by version.

Version 0:

Reviewer comments:

Reviewer #1

(Remarks to the Author)
This paper investigates the impact of electrical activation of the SNc/VTA on visual responses in the superior colliculus (SC). The authors find that pairing activation of the SNc/VTA with visual stimulation leads to an increase in the visual evoked potential (VEP) as well as spiking in the SC. A similar increase in the VEP was observed in anesthetized and freely moving animals. Injection of a D1-like dopamine receptor antagonist or a 5-HT1A receptor antagonist into the SC blocked potentiation of the VEP during pairing of visual stimulation with electrical activation of the SNc/VTA. The authors go on to show that pairing activation of the SNc/VTA with visual stimulation leads to an increase in dopamine release in the striatum as well as the NAcc during visual input. Importantly, this dopamine signal is blocked by blocking D1 receptors in the SC, suggesting that it originates in the SC.

Overall this is an interesting paper, which provides evidence that increased activation of the deeper layers of the SC enhances dopamine release following classical conditioning.

Comments:

1. I was surprised that injection of both dopamine and 5-HT antagonists into the SC blocked potentiation of VEPs. Where do the authors think the release of 5-HT is coming from? Or is it possible that SNc/VTA stimulation activates 5-HT fibers of passage or antidromically drives 5-HT terminals in the SNc/VTA?

2. The VEP reflects activation of many cells with its sign dependent on the location of sources and sinks relative to the position of the recording electrode. Interpretation of changes in the VEP is therefore complex. While the authors show that the increase in the negative component of the VEP is correlated with spiking, I would have liked to have also seen evidence for changes in spiking during the injection of dopamine and 5-HT antagonists.

3. Figure 5 and associated text in the Results is pretty confusing. Firstly, please define “FCV” when this first appears in the Results. Secondly, I don’t understand the color FCV plots in panels b and c. What is the y-axis and what does “Red” and “Ox” represent? Thirdly, I don’t see “a new striatal dopamine signal in response to the light, which had been absent at baseline” in Figure 5b. No baseline data are shown as far as I can tell.

Reviewer #2

(Remarks to the Author)
In this manuscript, Zhang et al. investigate the role of the superior colliculus (SC) in visual conditioning, where a neutral stimulus (light flashes) is paired with an unconditioned stimulus (US; SNr/VTA stimulation). Using in vivo recordings, fast-scan cyclic voltammetry, calcium imaging, and pharmacological manipulations in rats, the authors explore the mechanisms underlying this process. They provide evidence that the deep layers of the SC mediate the association between the conditioned stimulus (CS) and US, as indicated by increased activity following light flashes. This activity subsequently drives downstream dopamine responses, highlighting the SC’s integrative role in linking sensory inputs with reward-related neural circuits. Potentiation of SC responses, mediated by dopamine D1 and serotonin 5-HT1A receptors, is shown to

enhance dopamine release in the striatum, forming a feedback loop critical for associative learning. Importantly, blocking the SC's visual response potentiation abolishes dopamine responses to the CS, underscoring the SC's pivotal role in this process.
These findings reveal a feedback mechanism in which the SC not only mediates the association between the CS and US but also drives increased dopamine release in downstream regions, such as the striatum, through its interaction with the SNr/VTA. This dual role in classic conditioning positions the SC as both an integrator of sensory inputs and a driver of dopamine-mediated associative learning, providing new insights into how sensory and reward pathways interact. While the study's conclusions present intriguing ideas with potential significance for the field, certain aspects of the experimental design and data interpretation introduce limitations that should be addressed to fully realise its impact. Below, I outline key concerns, both major and minor, regarding the methodology, data presentation, and interpretation of results.

Major point:

1. Data Strength and Sample Size: Many interpretations rely on low-amplitude signals (e.g., small visual evoked potentials [VEPs] < 100 µV, calcium signals ~ 0.1% DF/F) or experiments with limited sample sizes, both of which pose challenges to statistical power and reproducibility. For instance:
a) While Figure 1 small negative VEPs (nVEPs) are confirmed by multi-unit activity (MUA) in Figure 2, the latter appears based on only one rat, raising concerns about generalizability.
b) While Figures 2 and 4 lack clear indications of N values, Extended Data Fig. 2 uses data from only one animal, and Figures 3 and 5 rely on N = 3 or 4 rats.
Interestingly, MUA recordings reveal stronger LFP/nVEP responses, aligning more closely with expected sensory-evoked responses. This raises the possibility that the weaker signals recorded with glass pipettes may reflect limitations of the recording method. Reproducing nVEP-based results using the MUA recording system, which appears more effective at capturing LFP signals, would enhance confidence in the findings. To further strengthen the conclusions, power analyses should be conducted to ensure adequate sample sizes, and additional animals should be included where needed. This would improve the robustness and reproducibility of the results.

2. Support for Conclusions: Some conclusions in the manuscript would benefit from stronger direct experimental evidence or clearer explanation. Specific examples include:
a) The statement: “The amplitude and persistence of the nVEP in the deep SC was positively correlated with dopamine neuron activity during novel visual stimulation” is not supported by recordings in the SNr/VTA or an explicit correlation analysis.
b) The results title, “…but consistently faster”, implies simultaneous dopaminergic and SC activity measurements, which are not provided. What is the evidence in Fig 1 supporting this statement?
c) The claim that the nVEP timing is "ideal" for driving dopamine responses is based solely on bicuculline disinhibition experiments in one rat, which limits generalisability.
d) The assertion: “The deep layers of the SC identify the salience of the visual CS” seems overstated, as salience appears to be externally imposed via SNr/VTA stimulation.
e) The statement that the results “characterise the eligibility trace for visual stimuli within SC” lacks clarity or supporting evidence, making it difficult for readers to follow the rationale behind the claim.
To improve the manuscript, the authors are encouraged to either revise these conclusions to better align with the presented evidence or provide additional explanation and analysis to clarify their claims.

3. Model and interpretation of salience: One limitation of the proposed model is that it does not acknowledge the critical role of SNr/VTA input in driving the association between the US and CS in the SC. The authors present the SC as the primary locus of associative learning, yet their data clearly demonstrate that dopamine release in the SC, triggered by SNr/VTA stimulation (the US), is essential for facilitating this association. Without this dopaminergic input, the SC likely would not associate the CS with the US. Specifically, the SNr/VTA input provides the salience signal (via dopamine) that enables plasticity in the SC when the CS arrives. This plasticity then allows the SC to drive enhanced dopamine responses to the CS through feedback to the SNr/VTA. While the SC plays an important integrative role in this process, it is not acting autonomously, as the current model might imply. The model should illustrate how the US drives dopamine release in the SC to enable the association, which subsequently leads to SC-driven feedback to the SNr/VTA during the CS. This clarification would strengthen the interpretation of their findings and more accurately reflect the SC's role as part of a broader circuit, rather than an independent driver of associative learning.

4. Behavioural relevance of SC-mediated dopamine release: Dopamine release observed following the CS is significantly smaller in amplitude compared to release driven by direct activation of the SNr/VTA. This raises questions about whether such a modest effect is sufficient to produce meaningful behavioural outcomes.

Minor points:

1. Terminology and Context:
• Some abbreviations are not introduced (e.g. SNr/VTA, FCV). 3rd sentence results: SNr/VTA. Should read: Substantia Nigra pars reticulata (SNr) Ventral tegmental area (VTA). Ideally, for the benefit of readers unfamiliar with sources of dopamine and conditioning, the involvement of SNr and VTA should have been introduced in the intro.
• While this concept is repeated across the manuscript, it is unclear what makes the 1-2s time interval “behaviourally meaningful”. Please give explicit examples of how the time windows are behaviourally relevant.

2. Methods and Analysis:
• Detailed descriptions of the analysis methods (e.g., normalization of dopamine release and calcium responses) are missing and should be included, as these would highly affect interpretation.
• Clarification needed in Figure 1d: The term "mean nVEP" is misleading when values before pairing are positive. What is being compared? Is it the minimum VEP? If so, is there a time limit after which you would not look for negative VEPs any longer, e.g. "minimum VEP within X ms"? If not, is it the VEP at specific time? When comparing nVEPs, if none, the value should be 0 not positive.
• Figure 4. Is the VEP calculated by subtracting the baseline? If not, it would be useful to know what was the effect on baseline activation before pairing too.

3. Figures and Data Presentation:
• Figure 1b: the positive response to visual stimulation seems much stronger than the negative response. Later in the manuscript this is attributed to the direct activation of superficial SC; however, this should be discussed at this point instead. The SC has a very high proportion of inhibitory neurons (up to 70%) in superficial layers, is that expected to affect the measured VEP? Since VEP is harder to interpret than neuronal firing, it would be helpful to have a discussion on origin of the VEP signal.
• Figure 1d indicates N = 5 animals in the main text and legend but shows 9 data points per bar in the graph.
• Figure 5c: The inset colour scheme is confusing, as it uses the same colours for different variables (nA and time).

4. Interpretations:
• Figure 1h: The presence of a negative VEP in the "-/-" condition is unexpected and should be addressed.
• The authors mention an increase in movement when whole-field light flash was paired with SNr/VTA stimulation. It is hard to envision how this could relate to reward prediction/expectation. Alternatively, an increase in activity in the SC could increase the probability of (somewhat behaviourally irrelevant) motor output by the SC. Would be useful to describe what type of movement is observed (e.g. running?) and what is the expected behavioural meaning of such movement.

5. Others:
• The title "US and CS is…" should read "US and CS are".

Reviewer #3

(Remarks to the Author)
The work demonstrates that a negative visually evoked potential (nVEP) emerges in the deep layers of the superior colliculus when visual stimulation is paired with stimulation of the SNc/VTA one second later. The authors show that this potentiation can also be induced using a similar protocol in freely moving rats receiving intracranial self-stimulation paired with a visual stimulus. They also show that the potentiation of the nVEP in the deep layers of the SC is blocked by application of dopamine D1 and serotonin 5-HT1A antagonists but not with a 5-HT2A antagonist. They also show that blocking D1 receptors in the SC decreases dopamine levels in the striatum and reduces dopaminergic axonal responses in the striatum. The authors conclude that neurons in the deep layers of the SC develop neuronal responses to a visual conditioned stimulus, which in turn drive the responses of dopamine neurons.
The work is relevant for the field of systems neuroscience and shows that visually driven conditioned responses emerge in the deep superior colliculus and provides evidence that responses of dopamine neurons to a conditioned stimulus decrease when the potentiation of the visual response in the deep layers of the SC is blocked.
Given the specific points raised below, some claims in the paper might need additional support, which I expect could feasible be obtained through further analysis of the existing data. The methodological approach is appropriate, and the methods should enable other researchers to replicate the results.
Specific points:
Related to figure 1:
1) Fig 1. It would be helpful to introduce the habituation of the responses earlier in the figure. This would help the reader understand why the values from -10 to -5 minutes are higher than those between -5 and 0 early on. Also, to support the choice of analysing the nVEP with respect to the baseline considering only the 5 minutes before the paring starts as stated in the methods ('Because the nVEP showed its minimum value at the last 5 min of baseline recording and its maximum value during the last 5 min of pairing, the difference of the average LFP between these two periods was defined as the amplitude of the nVEP (Fig. 1').
2) The distribution of the peak latencies for the nVEP across animals should be shown, and the standard deviation (e.g., 70 ms ± ?) should be reported.
3) Fig 1b. Do the upper and lower panels have the same bar scale? The scale in the upper panels look smaller than the scale in the lower panels.
4) Fig 1d. The statistical analysis should account for the repeated-measures design. Moreover, in some animals the protocol was applied twice. Are there any differences between the values corresponding to the first and the second protocol? The Effect of repeating the pairing protocol in the same animal and if possible taking into account the time that passed between the first pairing protocol and the second should be carefully analysed as if I understand correctly the work in figure 5 relies in analysing the effect of a drug application on a second pairing protocol, after the control was performed.

5) If the figure is plotting the nVEPs, then the y-axis label should read 'nVEP' instead of VEP. Same for other figure panels in the manuscript.

6) Fig. 1f lacks statistical analysis; only one example trace is provided to support the claim that 'nVEP potentiation can be induced through low pairing frequencies'.
Related to Figure 2:
1) In the mouse experiments, the MUA activity is not high in the first few trials, there are no spikes in the first few trials in panel d, and there is a low MUA response in the time window -10 to -5 (panel e, also missing units in y axis). However, the work in rats in figure 1 showed high nVEPs in the first few trials followed by habituation. Could the authors comment on the lack of MUA activity in the first few trials of the protocol in the deepSC?
2) Does the data in this figure correspond to N=1 animal? This seems a low number of animals.
3) Fig 2f shows the correlation between MUA and nVEP magnitude for one example channel. Could the authors please report the correlation coefficient for the rest of the channels? Is this high correlation representative of all the other channels? Is this result reproducible across animals?
Related to Figure 3:
1) The following statement is not supported by any data or figures: 'Moreover, following pairing, animals responded to the whole-field light flash with movement, consistent with others'observations in classical conditioning22. This contrasted with the absence of such movements in response to the light during baseline or prolonged extinction (> 10 min), suggesting they had learnt through pairing the significance of the light flash as a predictor of SNc/VTA reward delivery.'
2) In relation to the previous point, considering that activity in the deep SC can be linked to the generation of motor movements, could the authors please provide evidence that the correlation of the nVEP with motor movement in Supplementary figure 3, does not simply reflect motor related activity that would be present regardless of the application of the pairing protocol.
3) Is there a motor movement observed in the first presentation of the visual stimulus before the habituation of the visual responses? The correlation could be calculated for the difference in movement/nVEP for the pre-habituation period with the pre-pairing protocol.
Related to Figure 4.
1) The authors should state in the main text if the experiments were done in anesthetised rats.
2) It would be useful to present the traces observed with saline in panel 4c, in order to show that saline injection does not affect responses in any way (beyond the nVEP presented in panel 4b).
3) The authors should also comment on the effects of the drugs on the rest of the response. Panel 4c suggests that the 5HT1A antagonist injection might have a prolong effect beyond the nVEP. Could it have general inhibitory effect, instead of a specific effect on the nVEP?
4) The traces corresponding to the injection of ketanserin should be presented as Supplementary figures.
Related to Supplementary figure 4:
1) Does the data correspond to one animal? Is it rat or mouse. This information should be provided in the figure legend. To demonstrate that the pVEP in the deep layers is correlated with the neuronal activity in the superficial layers but not the nVEP, the cross-correlation should be calculated for several animals, and the peak and time of the cross-correlation shown.
2) The following statement should be revised: 'The positive deflection of the VEP (pVEP) in the deep layers of the SC, […] was also potentiated during the pairing protocol (≈ 30 to 40 ms latency; see Fig. 1c), but this effect did not persist during the extinction period […]. This indicates that the potentiated nVEP in the deep layers is not secondary to the potentiation of neuronal activity in the superficial layers.' First the authors do not provide any quantification or statistical analysis of the pVEP to show that it dos not persist during extinction, second the authors do not provide evidence that there is no potentiation of neuronal activity in the superficial layers of the SC.
3) The following statement: 'SCH23390 or WAY100635 diminished only the nVEP but not the pVEP during CS-US pairing (Fig. 4b)' is not supported by a figure, as the pVEP was not compared in Fig 4b. Please provide the supporting material for the statement.
Related to Figure 5.
1) Fig. 5b is missing y axis title.
2) Fig. 5c. Inset should separately show the traces for the control and the D1 antagonist injection.
3) Figure legend should state if the control corresponds to recordings performed in the same animal, and then the pairing protocol was repeated after the injection of the D1 antagonist injection. If this is the case, a more relevant control would have been to compare to saline injections in the SC and perform the same experimental sequence.

Other points:
1) The authors propose that 'the superior colliculus is the brain area that identifies the saliency of visual cues in classical conditioning and drives dopamine phasic activity.' The authors should consider that in the mouse 85% of retinal ganglion cells (RGCs) project to the SC, whereas in primates the SC receives input from about 15% of RGCs. As such, the rodent SC might play a more crucial role in classical conditioning that it might play in other mammals. Moreover, since the role of other visual areas remains undetermined and only a single visual stimulation protocol (lacking complex visual features) was tested, the authors may consider rephrasing this statement.
2) Some abbreviations are not defined upon first appearance, including fast-scan cyclic voltammetry (FCV) and SNc/VTA."

Version 1:

Reviewer comments:

Reviewer #2

(Remarks to the Author)
The authors have addressed most of my previous concerns and clarified several methodological and interpretative points. However, there are two issues that remain unclear:

Point 2.a — This point referred to a sentence related to Figure 1, where no DA was measured. The authors' rebuttal indicates that this statement was revised; however, in the revised manuscript, I could not identify where.
Point 2.b — The results subsection title and phrasing implying "consistently" faster responses still suggest a repeated or simultaneous measure of SC and dopamine neuron activity, which was not performed. The authors provide a reasonable explanation for the assumption that the response was faster than previously measured DA activity and stated in their response that they revised the title and text accordingly, but the title still appears unchanged in the revised version I received.

These are relatively minor points, raised to help prevent potential misinterpretation by readers. Aside from these points, I am happy with the revisions and supportive of publication once these clarifications are addressed.

Reviewer #3

(Remarks to the Author)
The authors have addressed all of my concerns.

Open Access This Peer Review File is licensed under a Creative Commons Attribution 4.0 International License, which permits use, sharing, adaptation, distribution and reproduction in any medium or format, as long as you give appropriate credit to the original author(s) and the source, provide a link to the Creative Commons license, and indicate if changes were made.
In cases where reviewers are anonymous, credit should be given to 'Anonymous Referee' and the source.
The images or other third party material in this Peer Review File are included in the article's Creative Commons license, unless indicated otherwise in a credit line to the material. If material is not included in the article's Creative Commons license and your intended use is not permitted by statutory regulation or exceeds the permitted use, you will need to obtain permission directly from the copyright holder.
To view a copy of this license, visit https://creativecommons.org/licenses/by/4.0/

Reviewer #1 (Remarks to the Author):

This paper investigates the impact of electrical activation of the SNc/VTA on visual responses in the superior colliculus (SC). The authors find that pairing activation of the SNc/VTA with visual stimulation leads to an increase in the visual evoked potential (VEP) as well as spiking in the SC. A similar increase in the VEP was observed in anesthetized and freely moving animals. Injection of a D1-like dopamine receptor antagonist or a 5-HT1A receptor antagonist into the SC blocked potentiation of the VEP during pairing of visual stimulation with electrical activation of the SNc/VTA. The authors go on to show that pairing activation of the SNc/VTA with visual stimulation leads to an increase in dopamine release in the striatum as well as the NAcc during visual input. Importantly, this dopamine signal is blocked by blocking D1 receptors in the SC, suggesting that it originates in the SC.

Overall this is an interesting paper, which provides evidence that increased activation of the deeper layers of the SC enhances dopamine release following classical conditioning.

Comments:

1. I was surprised that injection of both dopamine and 5-HT antagonists into the SC blocked potentiation of VEPs. Where do the authors think the release of 5-HT is coming from? Or is it possible that SNc/VTA stimulation activates 5-HT fibers of passage or antidromically drives 5-HT terminals in the SNc/VTA?
We thank the reviewer for raising the question regarding the source of 5-HT innervation in the superior colliculus (SC). The SC receives dense serotonergic input from the dorsal raphe nucleus, median raphe nucleus, raphe pontis, and from the contralateral periaqueductal gray (Beitz et al., 1986). Interestingly, a substantial proportion of 5-HT synaptic contacts in the SC are axo-axonic (Arce et al., 1995), which supports our hypothesis that 5-HT may play a key role in translating salient information to the deep layers of the SC.

We have addressed this issue in the manuscript '*In addition, all layers of the SC receive massive serotonergic projections from the raphe nuclei*[31,32]'.

2. The VEP reflects activation of many cells with its sign dependent on the location of sources and sinks relative to the position of the recording electrode. Interpretation of changes in the VEP is therefore complex. While the authors show that the increase in the negative component of the VEP is correlated with spiking, I would have liked to have also seen evidence for changes in spiking during the injection of dopamine and 5-HT antagonists.

We appreciate the reviewer's insightful comment regarding the interpretation of VEPs and the importance of spike recordings. Due to technical constraints, we were unable to reliably record spiking activity during local drug injections. Stable spike recordings require a consistent electrode–neuron relationship, which can be disrupted by the mechanical effects of local drug delivery. For this reason, we chose to use the LFP as a readout of superior colliculus activity, as it represents the summed local synaptic and spiking activity. Notably, only 5-HT antagonists and D1 antagonists, but not saline, altered the amplitude of the negative phase of the LFP, supporting the conclusion that both serotonin and dopamine contribute to this process.

3. Figure 5 and associated text in the Results is pretty confusing. Firstly, please define "FCV" when this first appears in the Results. Secondly, I don't understand the color FCV plots in panels b and c. What is the y-axis and what does "Red" and "Ox" represent? Thirdly, I don't see "a new striatal dopamine signal in response to the light, which had been absent at baseline" in Figure 5b. No baseline data are shown as far as I can tell.

Thank you for pointing out that the abbreviation "FCV" was not defined in the manuscript. We have now addressed this by defining it at its first mention.

In the colour plots, the y-axis represents the voltage of the scan, during which the electrode cycles between –0.4 V and 1.3 V. "Ox" denotes the oxidation potential for dopamine, and "Red" denotes the reduction potential. Following visual stimulation, a new purplish signal appears at the oxidation potential for dopamine, which reflects the newly evoked striatal dopamine signal; the baseline signal at this potential is yellowish.

We have added further clarification about 'Ox' and 'Red' in the legend of Figure 5, which we hope will make the figure and associated description clearer for readers.

Reviewer #2 (Remarks to the Author):

In this manuscript, Zhang et al. investigate the role of the superior colliculus (SC) in visual conditioning, where a neutral stimulus (light flashes) is paired with an unconditioned stimulus (US; SNr/VTA stimulation). Using in vivo recordings, fast-scan cyclic voltammetry, calcium imaging, and pharmacological manipulations in rats, the authors explore the mechanisms underlying this process. They provide evidence that the deep layers of the SC mediate the association between the conditioned stimulus (CS) and US, as indicated by increased activity following light flashes. This activity subsequently drives downstream dopamine responses, highlighting the SC's integrative role in linking sensory inputs with reward-related neural circuits. Potentiation of SC responses, mediated by dopamine D1 and serotonin 5-HT1A receptors, is shown to

enhance dopamine release in the striatum, forming a feedback loop critical for associative learning. Importantly, blocking the SC's visual response potentiation abolishes dopamine responses to the CS, underscoring the SC's pivotal role in this process.
These findings reveal a feedback mechanism in which the SC not only mediates the association between the CS and US but also drives increased dopamine release in downstream regions, such as the striatum, through its interaction with the SNr/VTA. This dual role in classic conditioning positions the SC as both an integrator of sensory inputs and a driver of dopamine-mediated associative learning, providing new insights into how sensory and reward pathways interact. While the study's conclusions present intriguing ideas with potential significance for the field, certain aspects of the experimental design and data interpretation introduce limitations that should be addressed to fully realise its impact. Below, I outline key concerns, both major and minor, regarding the methodology, data presentation, and interpretation of results.

Major point:

1. Data Strength and Sample Size: Many interpretations rely on low-amplitude signals (e.g., small visual evoked potentials [VEPs] < 100 μV, calcium signals ~ 0.1% DF/F) or experiments with limited sample sizes, both of which pose challenges to statistical power and reproducibility.

We appreciate the reviewer's important point regarding the strength of the signals and the sample sizes.

First, with respect to signal amplitude, while the VEPs we report are indeed small (<100 μV) and calcium signals modest (~0.1% ΔF/F), these small signals are nevertheless robust and reproducible, as they were recorded with well-established methods. Importantly, our analyses focus not solely on absolute amplitude, but on systematic changes relative to baseline or between conditions, which remain statistically significant despite the modest absolute magnitude.

Second, regarding sample sizes, we acknowledge that some experiments involve a limited number of animals, particularly in technically demanding protocols such as intracranial recordings with pharmacological manipulations (we also explain in more detail later). We emphasise that these were designed as within-subject experiments, in which each animal served as its own control, thereby increasing statistical power. In addition, we have used multiple convergent approaches (LFP, calcium imaging, FCV) that yield consistent results across modalities, further strengthening the reproducibility of our interpretations.

Finally, to address the reviewer's concern, we have clarified these points in the revised Methods.

For instance:
a) While Figure 1 small negative VEPs (nVEPs) are confirmed by multi-unit activity (MUA) in Figure 2, the latter appears based on only one rat, raising concerns about generalizability.

We thank the reviewer for highlighting the need for clearer labelling in Figure 2. To address this, we revised the figure caption and main text to explicitly state that the MUA recordings in Figure 2b and 2d are example traces from one animal, selected to illustrate typical response patterns, while the summarised data in Figure 2c and 2e represent the mean results across four animals. This distinction was not sufficiently clear in the original manuscript, and we appreciate the opportunity to improve readability. The consistency of the MUA patterns across all four animals supports the robustness of our findings, as evidenced by the statistical significance in Figure 2c and 2e.

b) While Figures 2 and 4 lack clear indications of N values, Extended Data Fig. 2 uses data from only one animal, and Figures 3 and 5 rely on N = 3 or 4 rats. Interestingly, MUA recordings reveal stronger LFP/nVEP responses, aligning more closely with expected sensory-evoked responses. This raises the possibility that the weaker signals recorded with glass pipettes may reflect limitations of the recording method. Reproducing nVEP-based results using the MUA recording system, which appears more effective at capturing LFP signals, would enhance confidence in the findings. To further strengthen the conclusions, power analyses should be conducted to ensure adequate sample sizes, and additional animals should be included where needed. This would improve the robustness and reproducibility of the results.

We have now clarified the N values (4 animals) for Figures 2 and 4 in the revised figure legends. We also acknowledge that Extended Data Fig. 2 includes data from a single animal; this was intended only as a proof-of-principle demonstration. The visual response in the superior colliculus after local disinhibition with bicuculline has been extensively characterised in our own and others' previous publications (Dommett et al., 2005, Science; Schulz et al., 2009, J Neurosci). Here, our aim was to illustrate that the latency of the nVEP induced by a rewarded visual stimulus is comparable to that observed in bicuculline-treated animals, thereby demonstrating that visual information is rapidly transmitted from superficial to deep layers, just as when the colliculus is locally disinhibited.

We agree that VEPs recorded with multi-unit electrode arrays are typically larger than those obtained with glass pipettes. As the reviewer suggests, this reflects methodological differences rather than inconsistencies in the biological responses

themselves. In our experiments, glass pipettes were deliberately chosen because they allow pharmacological manipulation at defined sites, which is not possible with MUA arrays. Despite their smaller amplitude, the signals we report (including pharmacological manipulations) are systematic, reproducible across animals, and consistent with the higher-amplitude responses recorded with MUA. We have clarified this methodological rationale in the revised Results and Discussion.

We also appreciate the concern regarding sample sizes. The smaller N in Figures 3 and 5 reflects the technical difficulty of these experiments. For behavioural recordings, animals were implanted with multiple electrodes (for VEP recording in the superior colliculus, stimulation of dopamine neurons, and grounding), each requiring precise placement, insertion at different depths and angles, and fixation with screws and dental cement. Animals then had to be trained to verify stimulation efficacy by demonstrating a clear relationship between current intensity and lever pressing. Although rat skulls are larger than those of mice, these procedures remain extremely challenging, involving multiple electrodes, a headpiece, screws, and dental cement. Each surgery lasted >6 hours, and the reported animals represent successes from more than 40 attempts. In these experiments, we observe the ethical principle of reduction, by using the minimum number of animals required to report a reliable observation.

Similarly, the FCV experiments were highly technically demanding. Dopamine release in the striatum is spatially heterogeneous, with “hotspots” that can only be detected if the electrode tip ($\approx$100 μm) is precisely aligned with an effective release site while simultaneously receiving visual input. This required identifying a release site by electrical stimulation to first prove it is a hotspot for dopamine release, and then to verify this hotspot is also aligned to visual input. To prevent habituation, visual stimuli could only be applied sparingly. These experiments are considered prohibitively difficult by many of our colleagues. Indeed, our clear results represent successful recordings after >40 attempts. For this reason, we complemented FCV with calcium imaging, which is a slightly less technically challenging approach. The consistency across multiple methodologies, all converging on the same functional conclusion, strengthens the robustness of our findings despite modest N in some cases.

Finally, we note that although N is not large in all cases, we performed power calculations based on the observed effect sizes and variability. As presented in the Results, all reported experiments achieved acceptable statistical power. Importantly, many of our comparisons are especially powerful because we use within-animal controls, thereby reducing variability and increasing sensitivity. To ensure transparency, we have also expanded the Methods section to provide details of the power analyses performed.

2. Support for Conclusions: Some conclusions in the manuscript would benefit from

stronger direct experimental evidence or clearer explanation. Specific examples include:
a) The statement: “The amplitude and persistence of the nVEP in the deep SC was positively correlated with dopamine neuron activity during novel visual stimulation” is not supported by recordings in the SNr/VTA or an explicit correlation analysis.

We thank the reviewer for this helpful comment. The statement regarding dopamine neuron activity refers specifically to our measurements of dopamine release in the striatum and calcium imaging of dopamine axons, which are the recordings we performed. We have now clarified this point in the revised manuscript to ensure our interpretation is precise and accurately reflects the level at which the recordings were obtained.

b) The results title, “...but consistently faster”, implies simultaneous dopaminergic and SC activity measurements, which are not provided. What is the evidence in Fig 1 supporting this statement?

We thank the reviewer for raising this important point. The title phrase “...but consistently faster” refers to the well-established latency of dopamine neuron responses during visual classical conditioning, which has been extensively characterised across species (rodent, monkey) and shown to occur at ~110 ms (e.g., Schultz et al., 1997, Science; Pan et al., 2005, J Neurosci). In contrast, the negative VEP we report in the deep SC has a latency of ~75 ms. Thus, although we did not record dopamine cell body activity and SC responses simultaneously, the difference in response latency between these well-characterised dopamine signals and the SC nVEP provides the basis for our statement that the SC response is consistently faster. We have revised the Results title and text to make this rationale clearer and to avoid any implication of simultaneous measurement.

c) The claim that the nVEP timing is "ideal" for driving dopamine responses is based solely on bicuculline disinhibition experiments in one rat, which limits generalisability.

We thank the reviewer for this valuable comment. The nVEP evoked by visual stimulation during local bicuculline disinhibition has been extensively characterised both by us in our prior studies and by other groups. As suggested, we have lowered the tone in the manuscript where it is now ‘Therefore, the timing of the nVEP generated in SC is *an excellent candidate* for driving the dopamine response during classical conditioning’

d) The assertion: “The deep layers of the SC identify the salience of the visual CS”

seems overstated, as salience appears to be externally imposed via SNr/VTA stimulation.

We thank the reviewer for this helpful point. We agree that in our paradigm, the unconditional salience is imposed via midbrain stimulation. Our data show that by comparing LFP responses in the superior colliculus with or without reward, the nVEP only emerges when reward (midbrain stimulation) follows the visual cue. This is why we originally stated that "the deep layers of the superior colliculus identify the salience of the visual CS."

To avoid overstating this claim, we have now revised the text to clarify that this occurs "*where the unconditional salience is imposed via midbrain stimulation*," in line with the reviewer's suggestion.

e) The statement that the results "characterise the eligibility trace for visual stimuli within SC" lacks clarity or supporting evidence, making it difficult for readers to follow the rationale behind the claim.

To improve the manuscript, the authors are encouraged to either revise these conclusions to better align with the presented evidence or provide additional explanation and analysis to clarify their claims.

We thank the reviewer for raising this important point. Our intention in using the term "eligibility trace" was to describe how the transient visual-evoked response in the superior colliculus can provide the necessary temporal window for reinforcement signals (e.g., midbrain stimulation) to act upon, consistent with the classical definition of eligibility traces in reinforcement learning. We were proposing that our study may be able to contribute to the study of the eligibility trace. We recognise that in the current version of the manuscript, this rationale was not sufficiently clear.

To address this, we have revised the relevant text to clarify that our results suggest that the nVEP provides a temporally precise signal that could serve as an eligibility trace for reinforcement, rather than claiming that we have comprehensively "characterised" this trace.

The current description is '*By measuring neuronal activity, we will be able to study the eligibility trace for visual stimuli in future, where the nVEP within the SC may provide a temporally precise signal that plays a critical role in the eligibility trace.*'

3. Model and interpretation of salience: One limitation of the proposed model is that it does not acknowledge the critical role of SNr/VTA input in driving the association between the US and CS in the SC. The authors present the SC as the primary locus of

associative learning, yet their data clearly demonstrate that dopamine release in the SC, triggered by SNr/VTA stimulation (the US), is essential for facilitating this association. Without this dopaminergic input, the SC likely would not associate the CS with the US. Specifically, the SNr/VTA input provides the salience signal (via dopamine) that enables plasticity in the SC when the CS arrives. This plasticity then allows the SC to drive enhanced dopamine responses to the CS through feedback to the SNr/VTA. While the SC plays an important integrative role in this process, it is not acting autonomously, as the current model might imply. The model should illustrate how the US drives dopamine release in the SC to enable the association, which subsequently leads to SC-driven feedback to the SNr/VTA during the CS. This clarification would strengthen the interpretation of their findings and more accurately reflect the SC's role as part of a broader circuit, rather than an independent driver of associative learning.

We thank the reviewer for this very insightful comment. One striking aspect of our results is that the superior colliculus (SC) begins to respond to a visual stimulus once it is paired with a reward that arrives ~1 s later. We agree that midbrain activation plays a critical role in enabling this process. However, previous immunohistochemical studies have shown that dopamine neurons in the SNc/VTA do not directly project to the SC, and that most dopaminergic innervation in the SC arises from the A13 cell group (Bolton et al., 2015, eLife). This suggests that a broader circuit, beyond a direct SNc/VTA–SC connection, must contribute to the plasticity we observe in the SC.

We have revised the legend of Figure 6 to clearly emphasise that the SC's role should be understood within the context of a wider integrative network, rather than as an isolated locus of associative learning. The legend title now is 'A model for how the deep layers of SC *integrate information of US and CS* and drive striatal dopamine release during stages of visual classical conditioning.'

4. Behavioural relevance of SC-mediated dopamine release: Dopamine release observed following the CS is significantly smaller in amplitude compared to release driven by direct activation of the SNr/VTA. This raises questions about whether such a modest effect is sufficient to produce meaningful behavioural outcomes.

We agree with the reviewer that dopamine release following the CS is smaller in amplitude than that observed with direct SNc/VTA stimulation. However, this is expected for two reasons. First, the visual stimulation we used was brief (10 ms) compared to the 500 ms SNc/VTA electrical stimulation, and second, visual stimulation selectively activates only a subset of dopamine neurons receiving visual inputs, whereas SNc/VTA electrical stimulation is designed to maximally recruit the population. Importantly, behavioural relevance does not necessarily require large dopamine transients. Previous studies have shown that relatively modest dopamine release can play critical roles in driving plasticity at corticostriatal synapses and influence

reinforcement learning. However, determining whether dopamine release alone is sufficient to drive the behavioural effects we observed is beyond the scope of the present study, though this is an important question that we are very interested in exploring in future work.

Minor points:

1. Terminology and Context:
• Some abbreviations are not introduced (e.g. SNr/VTA, FCV). 3rd sentence results: SNr/VTA. Should read: Substantia Nigra pars reticulata (SNr) Ventral tegmental area (VTA). Ideally, for the benefit of readers unfamiliar with sources of dopamine and conditioning, the involvement of SNr and VTA should have been introduced in the intro.
• While this concept is repeated across the manuscript, it is unclear what makes the 1-2s time interval "behaviourally meaningful". Please give explicit examples of how the time windows are behaviourally relevant.

We have addressed these two issues as suggested.

To address the behaviourally meaningful concept as suggested, we have expanded our discussion by adding '*In addition, as demonstrated, applying rewarding electrical stimulation before the visual stimulation did not evoke an nVEP in the deep layers of the SC, further supporting our conclusion that the SC responds to the CS-US association only when the US is applied at behaviourally meaningful intervals.*'

2. Methods and Analysis:
• Detailed descriptions of the analysis methods (e.g., normalization of dopamine release and calcium responses) are missing and should be included, as these would highly affect interpretation.

Thanks for pointing this out. We have further clarified these in the figure legend.

• Clarification needed in Figure 1d: The term "mean nVEP" is misleading when values before pairing are positive. What is being compared? Is it the minimum VEP? If so, is there a time limit after which you would not look for negative VEPs any longer, e.g. "minimum VEP within X ms"? If not, is it the VEP at specific time? When comparing nVEPs, if none, the value should be 0 not positive.

Thanks for pointing this out. We have clarified this in the figure legend.

• Figure 4. Is the VEP calculated by subtracting the baseline? If not, it would be useful to know what was the effect on baseline activation before pairing too.

We thank the reviewer for highlighting this point. The VEPs are calculated by subtracting the baseline from the control group.

3. Figures and Data Presentation:

• Figure 1b: the positive response to visual stimulation seems much stronger than the negative response. Later in the manuscript this is attributed to the direct activation of superficial SC; however, this should be discussed at this point instead. The SC has a very high proportion of inhibitory neurons (up to 70%) in superficial layers, is that expected to affect the measured VEP? Since VEP is harder to interpret than neuronal firing, it would be helpful to have a discussion on origin of the VEP signal.

We thank the reviewer for this helpful suggestion. We have now addressed this point in the manuscript: *“Notably, while the nVEP is likely driven by local spike activity in the deep layers of the SC, the positive phase of the VEP at ~30 ms is more likely attributable to spike activity in the superficial layers of the SC, whose neurons project to the deep layers.”*

We agree that the high proportion of inhibitory neurons in the superficial SC is an important consideration for interpreting VEPs. However, a detailed dissection of how inhibitory circuits contribute to the VEP signal is beyond the scope of the present study. We have noted this limitation.

• Figure 1d indicates N = 5 animals in the main text and legend but shows 9 data points per bar in the graph.

We thank the reviewer for pointing this out. We realise that our labelling was not sufficiently clear. Each blue dot in Figure 1d represents the average of three nVEPs recorded across the 30-minute session for the five animals, rather than a single trial. This is why nine data points are shown per group. We have revised the figure legend to clarify this distinction.

• Figure 5c: The inset colour scheme is confusing, as it uses the same colours for different variables (nA and time).

The different colours represent dopamine release after visual stimulation and electrical stimulation. We have modified the figure to make it clearer and further explained this in the figure legend as suggested.

4. Interpretations:
• Figure 1h: The presence of a negative VEP in the "-/-" condition is unexpected and should be addressed.

We also find this interesting and have addressed this in our manuscript.

'The -/- group, in which the recording site was not expected to receive either visual or dopamine input, showed a slightly higher response than the other two control groups, though still much smaller than the paired condition.'

• The authors mention an increase in movement when whole-field light flash was paired with SNr/VTA stimulation. It is hard to envision how this could relate to reward prediction/expectation. Alternatively, an increase in activity in the SC could increase the probability of (somewhat behaviourally irrelevant) motor output by the SC. Would be useful to describe what type of movement is observed (e.g. running?) and what is the expected behavioural meaning of such movement.

The movement is increasing the speed of moving, i.e., running. A similar observation has been reported in other studies on classical conditioning.

5. Others:
• The title "US and CS is..." should read "US and CS are".
We have addressed this. Thanks!

Reviewer #3 (Remarks to the Author):

The work demonstrates that a negative visually evoked potential (nVEP) emerges in the deep layers of the superior colliculus when visual stimulation is paired with stimulation of the SNc/VTA one second later. The authors show that this potentiation can also be induced using a similar protocol in freely moving rats receiving intracranial self-stimulation paired with a visual stimulus. They also show that the potentiation of the nVEP in the deep layers of the SC is blocked by application of dopamine D1 and serotonin 5-HT1A antagonists but not with a 5-HT2A antagonist. They also show that blocking D1 receptors in the SC decreases dopamine levels in the striatum and reduces dopaminergic axonal responses in the striatum. The authors conclude that neurons in the deep layers of the SC develop neuronal responses to a visual conditioned stimulus, which in turn drive the responses of dopamine neurons.

The work is relevant for the field of systems neuroscience and shows that visually driven conditioned responses emerge in the deep superior colliculus and provides evidence that responses of dopamine neurons to a conditioned stimulus decrease when the potentiation of the visual response in the deep layers of the SC is blocked.
Given the specific points raised below, some claims in the paper might need additional support, which I expect could feasible be obtained through further analysis of the existing data. The methodological approach is appropriate, and the methods should enable other researchers to replicate the results.
Specific points:
Related to figure 1:
1) Fig 1. It would be helpful to introduce the habituation of the responses earlier in the figure. This would help the reader understand why the values from -10 to -5 minutes are higher than those between -5 and 0 early on. Also, to support the choice of analysing the nVEP with respect to the baseline considering only the 5 minutes before the paring starts as stated in the methods ('Because the nVEP showed its minimum value at the last 5 min of baseline recording and its maximum value during the last 5 min of pairing, the difference of the average LFP between these two periods was defined as the amplitude of the nVEP (Fig. 1').

Thanks for this suggestion. We have addressed the habituation in our manuscript.

'Closer inspection of the VEPs throughout the experimental protocol (Fig. 1d; N = 5 animals) revealed that the nVEP gradually decreased during the baseline period, when light was applied alone, reflecting habituation of the visual response in the superior colliculus.'

2) The distribution of the peak latencies for the nVEP across animals should be shown, and the standard deviation (e.g., 70 ms ± ?) should be reported.

Thanks for this. We have reported this '*76.16 ± 4.02 ms; mean ± SD*' in our manuscript.

3) Fig 1b. Do the upper and lower panels have the same bar scale? The scale in the upper panels look smaller than the scale in the lower panels.

We thank the reviewer for this careful observation. Yes, both the upper and lower panels in Figure 1b use the same scale bar. The apparent difference arises because in the lower panels, the traces are shown after subtraction of the baseline recordings, which makes the light artefact appear larger.

4) Fig 1d. The statistical analysis should account for the repeated-measures design. Moreover, in some animals the protocol was applied twice. Are there any differences

between the values corresponding to the first and the second protocol? The Effect of repeating the pairing protocol in the same animal and if possible taking into account the time that passed between the first pairing protocol and the second should be carefully analysed as if I understand correctly the work in figure 5 relies in analysing the effect of a drug application on a second pairing protocol, after the control was performed.

We thank the reviewer for this useful suggestion. In Figure 1d, the statistical analysis was performed using a repeated-measures design, and the significant differences reported reflect results across the five animals included.

With respect to protocol order, we randomized the application of control and experimental protocols wherever possible (e.g., varying the intervals between visual and electrical stimulation, and in some cases with drug injections as in Fig. 4). Across these experiments, the results were highly robust, and we did not observe systematic differences depending on whether the control protocol was applied first or second.

For the experiments in Figure 5, we applied the control protocol first, as the technical difficulty of dopamine recordings necessitated confirming that the recording site was correctly aligned to the visual inputs before proceeding with drug application. We maintained consistent intervals between the two paradigms across all animals. In all cases where the recording site was aligned to the visual input, i.e., when dopamine activity following visual stimulation was observed, the local application of dopamine antagonists in the superior colliculus reliably diminished the dopamine responses, supporting the reproducibility of our findings.

5) If the figure is plotting the nVEPs, then the y-axis label should read ‘nVEP’ instead of VEP. Same for other figure panels in the manuscript.

Thanks for the suggestion. We have changed this in our Figures 1, 2, 3, 4.

6) Fig. 1f lacks statistical analysis; only one example trace is provided to support the claim that ‘nVEP potentiation can be induced through low pairing frequencies’.
Related to Figure 2:
1) In the mouse experiments, the MUA activity is not high in the first few trials, there are no spikes in the first few trials in panel d, and there is a low MUA response in the time window -10 to -5 (panel e, also missing units in y axis). However, the work in rats in figure 1 showed high nVEPs in the first few trials followed by habituation. Could the authors comment on the lack of MUA activity in the first few trials of the protocol in the deepSC?

We thank the reviewer for these helpful observations.

For Figure 1f, the panel shown is a representative trace from 5 independent repeats. In all cases, the VEP traces evoked by 10 s and 30 s interval pairings were nearly identical,

consistent with the conclusion that low-frequency pairings can also induce nVEP potentiation. Because this comparison did not reveal significant differences and was not central to our main conclusions, we chose to present a representative trace. To clarify this, we have revised the figure legend to explicitly note that the trace shown is representative of 5 replicates and that the similarity across trials was consistent.

Regarding the MUA activity in mouse experiments (Figure 2), we agree that activity in the first few trials appears lower compared to the rat nVEP data in Figure 1. One explanation is that the relationship between MUA activity and nVEP amplitude may be nonlinear, such that relatively small changes in spiking can still generate detectable changes in nVEP. Another explanation is species differences, since MUA recordings were performed in mice whereas LFP recordings were performed in rats. It is possible that naïve salient events elicit weaker initial responses in mice than in rats, perhaps reflecting differences in attentional processing across species. Importantly, despite these differences, both MUA and nVEP recordings show a consistent trend of habituation during the first 10 minutes of the protocol. We have clarified this point in the Results to make the methodological and interpretive context more transparent.

'However, the habituation of spike activity was less prominent than that of the nVEP, which may reflect both the nonlinear relationship between spike activity and field potentials, as well as species differences in the encoding of naïve salient responses between mice and rats.'

2) Does the data in this figure correspond to N=1 animal? This seems a low number of animals.

We thank the reviewer for pointing this out. We realised our description was not sufficiently clear. The recordings shown were repeated in 4 animals, not a single case. We have clarified this in the revised manuscript to avoid confusion.

3) Fig 2f shows the correlation between MUA and nVEP magnitude for one example channel. Could the authors please report the correlation coefficient for the rest of the channels? Is this high correlation representative of all the other channels? Is this result reproducible across animals?

The data demonstrated in Fig 2f is the combined recordings from 7 channels of 4 animals. We have clarified this.

Related to Figure 3:
1) The following statement is not supported by any data or figures: 'Moreover, following pairing, animals responded to the whole-field light flash with movement, consistent

with others'observations in classical conditioning22. This contrasted with the absence of such movements in response to the light during baseline or prolonged extinction (> 10 min), suggesting they had learnt through pairing the significance of the light flash as a predictor of SNc/VTA reward delivery.'

We thank the reviewer for highlighting this. The movement data supporting this statement are shown in Extended Data Fig. 3. We have now made sure to explicitly reference this figure in the main text so that the link between the behavioural observation and the supporting data is clear.

2) In relation to the previous point, considering that activity in the deep SC can be linked to the generation of motor movements, could the authors please provide evidence that the correlation of the nVEP with motor movement in Supplementary figure 3, does not simply reflect motor related activity that would be present regardless of the application of the pairing protocol.

We thank the reviewer for raising this important point. The movement data shown in Extended Data Fig. 3 reflect the difference in movement speed during pairing compared to baseline. In baseline conditions, presentation of the light stimulus alone did not evoke movement, whereas following pairing, the same light stimulus consistently elicited movement, consistent with its acquired predictive value for reward. This demonstrates that the observed correlation between nVEP and movement does not simply reflect motor-related activity that would occur independently of the pairing protocol, but instead reflects behaviour contingent on the reward association.

3) Is there a motor movement observed in the first presentation of the visual stimulus before the habituation of the visual responses? The correlation could be calculated for the difference in movement/nVEP for the pre-habituation period with the pre-pairing protocol.

We thank the reviewer for this thoughtful suggestion. In our experiments, we did not observe movements in response to the very first presentation of the visual stimulus prior to habituation. One possible explanation is that the animals had previously been tested for the optical reward current in the same behavioural chamber, during which light cues may have been presented. This prior exposure could reduce the likelihood of a naïve motor response to the initial visual stimulus.

We agree that it would be valuable to explicitly test whether naïve animals exhibit movement responses during the pre-habituation period and to calculate correlations between nVEP and movement under those conditions. However, this comparison is beyond the scope of the current study.

Related to Figure 4.
1) The authors should state in the main text if the experiments were done in anesthetised rats.

Yes, these experiments were carried out in anaesthetised rats, and we have added this information to the figure legend.

2) It would be useful to present the traces observed with saline in panel 4c, in order to show that saline injection does not affect responses in any way (beyond the nVEP presented in panel 4b).

We thank the reviewer for this helpful suggestion. As requested, we have now included the saline traces in the new Extended Data Figure 4 to demonstrate that saline injection does not affect nVEP. This addition makes it clearer that the effects reported in Figure 4 are specific to the D1 and 5-HT1A manipulations.

3) The authors should also comment on the effects of the drugs on the rest of the response. Panel 4c suggests that the 5HT1A antagonist injection might have a prolong effect beyond the nVEP. Could it have general inhibitory effect, instead of a specific effect on the nVEP?

We agree with the reviewer that the 5-HT1A antagonist may exert a more general modulatory effect beyond the nVEP, as suggested by the prolonged response observed in panel 4c. While our data demonstrate a clear and reproducible effect on the nVEP, we cannot exclude the possibility of broader inhibitory actions.

In discussion we addressed '*In addition, the 5-HT1A antagonist could also exert effects beyond the nVEP itself, as suggested by the prolonged responses. This raises the possibility that the drug may act through a broader inhibitory or modulatory influence on local network activity within the superior colliculus, rather than exclusively targeting the nVEP*'.

4) The traces corresponding to the injection of ketanserin should be presented as Supplementary figures.

As suggested by the reviewer, we have also included this figure in our Extended Data Fig. 4.

Related to Supplementary figure 4:
1) Does the data correspond to one animal? Is it rat or mouse. This information should be provided in the figure legend. To demonstrate that the pVEP in the deep layers is

correlated with the neuronal activity in the superficial layers but not the nVEP, the cross-correlation should be calculated for several animals, and the peak and time of the cross-correlation shown.

We thank the reviewer for this insightful comment. The trace shown was recorded from one rat, and we have obtained similar recordings in both rats and mice. This example is included to illustrate the potential contribution of superficial layer neuronal activity to the deep layer LFP. The positive phase of the LFP recorded in the deep superior colliculus likely reflects the summation of inputs from the superficial layers rather than locally generated activity. Consistent overlap in timing, but not in amplitude, was observed between superficial and deep-layer signals at this recording site.

Accordingly, we show one representative trace to indicate where the positive component may arise. As the primary focus of the present study is on the negative component of the VEP. These details have been clarified in the revised figure legend.

2) The following statement should be revised: 'The positive deflection of the VEP (pVEP) in the deep layers of the SC, [...] was also potentiated during the pairing protocol (≈ 30 to 40 ms latency; see Fig. 1c), but this effect did not persist during the extinction period [...]. This indicates that the potentiated nVEP in the deep layers is not secondary to the potentiation of neuronal activity in the superficial layers.' First the authors do not provide any quantification or statistical analysis of the pVEP to show that it dos not persist during extinction, second the authors do not provide evidence that there is no potentiation of neuronal activity in the superficial layers of the SC.

We thank the reviewer for this insightful suggestion. We have revised the manuscript and removed the statement regarding the non-persistence of the pVEP, as this is not the core focus of the study. We agree that, even with a full analysis of neuronal activity across the superficial layers of the superior colliculus, it would not be practical to definitively demonstrate the absence of potentiation in these neurons. We appreciate the reviewer's helpful feedback, which has improved the focus and clarity of the manuscript.

3) The following statement: 'SCH23390 or WAY100635 diminished only the nVEP but not the pVEP during CS-US pairing (Fig. 4b)' is not supported by a figure, as the pVEP was not compared in Fig 4b. Please provide the supporting material for the statement.

We thank the reviewer for pointing this out. To ensure the manuscript accurately reflects our data, we have revised the statement regarding the pVEP. While the pVEP can provide insight into how the deep layers of the superior colliculus identify salient visual information, comparing pVEP amplitudes across animals is less convincing when

results are not statistically significant in unpaired t-tests, particularly given that the pVEP amplitude is smaller than that of the nVEP in many groups.

In the revised analysis, we focused on within-animal comparisons (saline vs. SCH23390), where the more powerful paired t-test confirms the lack of significant difference more reliably (Extended Data Fig. 4). The Results section has also been updated accordingly to reflect this change.

Related to Figure 5.
1) Fig. 5b is missing y axis title.

We thank the reviewer for pointing this out. We have revised Figure 5 and added the missing y-axis title in panel 5b. This updated figure also incorporates other adjustments, which are described in the revised manuscript.

2) Fig. 5c. Inset should separately show the traces for the control and the D1 antagonist injection.

We thank the reviewer for this helpful suggestion. To improve clarity, we have revised the figure and moved the inset to panel 5b. This allows the voltammograms corresponding to visual and electrical stimulations to be shown more clearly.

3) Figure legend should state if the control corresponds to recordings performed in the same animal, and then the pairing protocol was repeated after the injection of the D1 antagonist injection. If this is the case, a more relevant control would have been to compare to saline injections in the SC and perform the same experimental sequence.

We thank the reviewer for this helpful suggestion. Saline injections were indeed used as the control group, and we have now clarified this explicitly in the figure legend.

Other points:
1) The authors propose that 'the superior colliculus is the brain area that identifies the saliency of visual cues in classical conditioning and drives dopamine phasic activity.' The authors should consider that in the mouse 85% of retinal ganglion cells (RGCs) project to the SC, whereas in primates the SC receives input from about 15% of RGCs. As such, the rodent SC might play a more crucial role in classical conditioning that it might play in other mammals. Moreover, since the role of other visual areas remains undetermined and only a single visual stimulation protocol (lacking complex visual features) was tested, the authors may consider rephrasing this statement.

We thank the reviewer for raising this important point. We agree that the superior colliculus (SC) receives a substantially higher proportion of retinal ganglion cell inputs

in rodents compared to primates, and therefore the SC may play a relatively more prominent role in visual processing and associative learning in rodents. We also acknowledge that the contribution of other visual areas to this process remains to be determined, and that our experiments employed a single visual stimulation protocol lacking complex visual features. To address this, we have rephrased the statement in the manuscript to more accurately reflect our findings:

"Our data suggest that, in rodents, the superior colliculus is a critical site for integrating the salience of visual cues during classical conditioning and for contributing to phasic dopamine activity. However, given species differences in retinal input to the SC, as well as the limited range of visual protocols tested here, further work will be required to assess the generality of these findings across species and in relation to other visual brain areas."

2) Some abbreviations are not defined upon first appearance, including fast-scan cyclic voltammetry (FCV) and SNc/VTA."

We have corrected this. Thanks!

Reviewer #2 (Remarks to the Author):

The authors have addressed most of my previous concerns and clarified several methodological and interpretative points. However, there are two issues that remain unclear:

Point 2.a — This point referred to a sentence related to Figure 1, where no DA was measured. The authors' rebuttal indicates that this statement was revised; however, in the revised manuscript, I could not identify where.

We thank the reviewer for pointing this out. We have modified the sentence to read:

'Therefore, the amplitude and persistence of the nVEP in the deep SC increased with the number of rewarded visual stimulations and persisted beyond reward delivery,'

to avoid confusion that might be introduced.

Point 2.b — The results subsection title and phrasing implying "consistently" faster responses still suggest a repeated or simultaneous measure of SC and dopamine neuron activity, which was not performed. The authors provide a reasonable explanation for the assumption that the response was faster than previously measured DA activity and stated in their response that they revised the title and text accordingly, but the title still appears unchanged in the revised version I received.

We thank the reviewer for pointing this out. We have removed 'but consistently faster', and the subsection title now reads

'SC responds to conditioned visual stimuli like dopamine neurons'

These are relatively minor points, raised to help prevent potential misinterpretation by readers. Aside from these points, I am happy with the revisions and supportive of publication once these clarifications are addressed.
We thank the reviewer for their support.

Reviewer #3 (Remarks to the Author):

The authors have addressed all of my concerns.

We thank the reviewer for their support.
